# Reproducibility of temporally evolving seizure patterns and network connectivity in focal epilepsy

**DOI:** 10.3389/fneur.2025.1617317

**Published:** 2025-11-11

**Authors:** Yaohong Wei, Wei Zhang, Sinclair Xingzhou Liu, Biao Han, Qi Chen

**Affiliations:** 1 Center for Studies of Psychological Application, South China Normal University, Guangzhou, China; 2 School of Psychology, South China Normal University, Guangzhou, China; 3 Department of Neurology, Beijing Tsinghua Changgung Hospital, School of Clinical Medicine, Tsinghua University, Beijing, China

**Keywords:** stereotactic EEG, epileptogenic zone, low-voltage fast activity, reproducibility, waveforms, power spectra, functional connectivity

## Abstract

Clinical semiology, waveform patterns, frequency band characteristics, neuronal spiking activity, and connectivity network dynamics exhibit notable reproducibility across seizures in the same patient. However, whether these reproducible features distinctly delineate the epileptogenic zone (EZ), propagation zone (PZ), and non-involved zone (NIZ) and the underlying temporal dynamics remain unclear. In this study, we included 14 patients with focal epilepsy characterized by low-voltage fast activity (LVFA) and with pre-LVFA patterns of rhythmic spikes and/or burst of polyspikes. We examined the reproducibility of raw signals, power spectra, and connectivity patterns across multiple seizures during the interictal, pre-LVFA, and ictal periods. During the ictal phase, raw signals and power spectra in both the high (gamma and ripple) and low (delta) frequency bands demonstrated greater reproducibility in EZ than PZ. Since the high and low frequency bands during seizure onsets have been associated with fast inhibitory interneuron activity and principal neuron activity, respectively, the present results imply that activity of different neuronal populations in the EZ may remain stable across seizures. More importantly, during the pre-LVFA period, the reproducibility of beta band power spectra in EZ, the connectivity patterns within EZ and between EZ and PZ could already discriminate the EZ from the PZ prior to the LVFA onset. Taken together, the reproducibility of raw signals and power spectra in high and low frequency bands during the ictal period could help to improve diagnostic precision of EZ, and the reproducibility of pre-LVFA beta band power spectra, the connectivity within EZ and between EZ and PZ would aid in predicting seizure onsets.

## 1 Introduction

Epilepsy affects over 70 million individuals worldwide, with approximately one-third suffering from drug-resistant epilepsy (1). These patients can achieve seizure freedom if the epileptogenic zone (EZ)—the site of seizure onset and primary organization—is precisely identified and surgically removed (2). Stereotactic electroencephalography (SEEG) is commonly used in combination with other non-invasive diagnostic tools to localize the EZ (3).

Low-voltage fast activity (LVFA), typically observed in the beta to gamma frequency range (14–100 Hz) during SEEG (4), is frequently used as a biomarker to localize the epileptogenic zone (EZ) (5, 6). It is the most common seizure onset pattern, occurring in more than 80% of focal epileptic seizures (7). Although the presence of LVFA has been associated with better post-surgical outcomes compared to other onset patterns (8), it may not serve as a fully reliable biomarker. For example, Michalak et al. evaluated the prognostic value of various seizure onset patterns and found that 44% of patients with LVFA onset (including herald spike, polyspike, and burst followed LVFA) achieved seizure freedom 2 years after surgery (9). Furthermore, LVFA often propagates beyond the EZ (4), and studies have reported no significant differences in its frequency or onset timing between EZ and propagation zone (PZ) (10). These limitations underscore the need for additional biomarkers—beyond the characteristics of LVFA in a single seizure—to enhance the accuracy of EZ localization.

In a given patient, clinical semiology and underlying pathophysiological activity is typically consistent across seizures (11). Previous studies had demonstrated that the reproducibility across seizures could serve as a potential criterion, specifically, ictal core areas showed greater reproducibility in neuronal firing patterns compared to the ictal penumbra (12). Additionally, reproducible ictal waveform pattern (13, 14) and the frequency characteristics of fast activity (6) recorded across seizures at the same electrode sites were demonstrated. However, it remains unclear whether the temporally evolving reproducibility of waveforms and frequency characteristics can differentiate EZ from PZ. Moreover, seizure activity is often accompanied by other patterns beyond LVFA (7), such as pre-LVFA spikes and suppression of slow frequencies, which were identified as the “fingerprint” of the EZ along with LVFA (10). The reproducibility of these accompanying patterns is not yet fully understood.

Moreover, focal epilepsy is commonly viewed as a network disorder involving large-scale brain networks for seizure generation and propagation (15), while other perspectives suggest it follows a self-organizing model where localized neuronal activity within EZ drives seizure persistence and termination (16). EEG ictal connectivity is reproducible across seizures (17) and often propagates along stereotyped pathways (13, 18). However, it remains unclear whether this reproducibility reflects the entire epileptic network or is primarily confined to the EZ. In summary, we hypothesize that lower reproducibility in seizure-related regions compared to the non-involved zone (NIZ) reflects greater variability in seizure dynamics across seizures. Conversely, higher reproducibility in seizure-related regions relative to the NIZ suggests more stereotyped spatiotemporal patterns of seizure activity within patients. Importantly, we further hypothesize that between-seizure reproducibility differences within seizure-related regions—specifically between the EZ and the PZ—may provide additional information for delineating the EZ, offering a potential biomarker for localizing epileptogenic tissue.

To address these, we aimed to determine which specific reproducible characteristics can differentiate the EZ from the PZ. We compared the reproducibility of waveforms, power spectra, and connectivity patterns during the interictal, pre-LVFA and ictal periods across four seizures in 14 patients with focal epilepsy who have been seizure-free or almost seizure-free after resection surgery. A semi-automated method was employed to estimate the onset of LVFA in each electrode, ensuring alignment across seizures. Reproducibility of waveforms and corresponding frequency spectra, including delta, theta, alpha, beta, gamma, ripple, and fast ripple bands, respectively, was calculated using nonlinear correlation and Pearson correlation (19). Additionally, the reproducibility of connectivity patterns within- and between- region across seizures was evaluated with representational similarity analysis (20).

## 2 Materials and methods

### 2.1 Patient selection and SEEG recordings

We included 14 patients with drug-resistant epilepsy who underwent preoperative SEEG evaluation at Shanghai Deji Hospital between 2017 and 2019. The study was approved by the South China Normal University review board, and all patients provided written informed consent. Inclusion criteria were: (i) tailored resection guided by SEEG; (ii) at least four SEEG seizures with LVFA onset; and (iii) over 1 year for postoperative follow-up with seizure-free or almost seizure-free outcomes. Patient selection was independent of epilepsy type. Patients exhibited pre-LVFA patterns of rhythmic spikes and/or bursts of polyspikes. Pre-LVFA patterns and clinical profiles of the patients were summarized in Table 1.

**Table 1:** Clinical characteristics of patients.

Patient	Gender	AAO (y)	AASEEG (y)	MRI Lesion	Surgery	Pathology	Follow-up duration(m)	Outcomes (Engel)	Pre-LVFA patterns
1	F	39	47	Right amygdala and anterior hippocampus	Right anterior temporal lobe, anterior insula, and posterior orbitofrontal cortex	Nonsepcific	15	I	RS
2	F	14	29	Negative	Right medial frontal lobe and posterior orbitofrontal cortex	Nonsepcific	45	I	RS
3	F	4	19	Right hippocampus	Right anterior temporal lobe and hippocampus	HS	81	I	RS + RSBPs
4	M	1	27	Right temporal lobe and parietal lobe	Right temporal lobe and anterior insula	encephalomacacia	56	II	BPs
5	F	25	30	Negative	Left frontal lobe medial	FCD	79	I	RSBPs
6	F	9	21	Negative	Right lateral parietal lobe	FCD	79	I	RSBPs
7	F	15	32	Negative	Right insula and lateral central cortex	Nonsepcific	68	I	RSBPs
8	M	2	9	Bilateral occipital lobe	Right lateral and medial occipital lobe	encephalomacacia	67	II	RS + BPs
9	M	8	14	Right temporal lobe	Right anterior temporal lobe and anterior insula	HS	56	I	RS + RSBPs
10	M	27	31	Left amygdala and hippocampus	Left anterior temporal lobe and anterior insula	HS	60	I	RS + RSBPs
11	M	9	17	Left parietal lobe	Left lateral parietal lobe	FCD	87	I	RS
12	F	3	8	Negative	Right orbitofrontal cortex	FCD	93	I	BPs + RS + RSBPs
13	F	8	12	Negative	Left lateral central cortex	FCD	56	I	BPs + RSBPs
14	F	24	26	Right hippocampus and anterior temporal lobe	Right anterior temporal lobe and hippocampus	HS	78	I	RS

AAO, age at onset of the epilepsy; AASEEG, age at the time of the SEEG; BPs, burst of polyspikes; FCD, focal cortical dysplasia; HS, hippocampal sclerosis; RS, rhythmic spikes; RSBS, rhythmic spikes followed by burst of polyspikes; y, years; m, months.

In addition, we analyzed one extra patient who underwent a failed initial resection (Engel III outcome) followed by a successful second surgery (Engel I outcome). This case was used as an independent validation and was not included in the primary cohort analyses.

Pre-surgical assessments included medical history, neuropsychological evaluations, video EEG and magnetic resonance imaging (MRI). SEEG was performed following a comprehensive analysis of these clinical and pre-surgical data. Intracranial electrodes (10–15 leads, 2 mm length, 0.8 mm diameter, 1.5 mm spacing) were implanted using the ROSA robotic system according to Talairach’s stereotactic method. Electrode placement was determined based on hypotheses regarding the EZ localization from non-invasive assessments. On average, 15.6 electrodes were implanted per patient. SEEG signals were recorded with a Nihon-Kohden system, using a sampling rate of 2000 Hz and a 0.016–600 Hz band-pass filter.

### 2.2 Definition of regions and periods of interest

For each patient, three regions were defined based on LVFA presence and resection area: (i) EZ, regions within the resection zone exhibiting consistent LVFA across seizures; (ii) PZ, regions outside the resection zone with consistent LVFA across seizures; and (iii) NIZ, regions not involved in seizures. Figure 1 illustrates zone definitions and corresponding signals. The spatial distribution of each patients’ electrodes was illustrated in Supplementary Figures 1–14.

**Figure 1:**
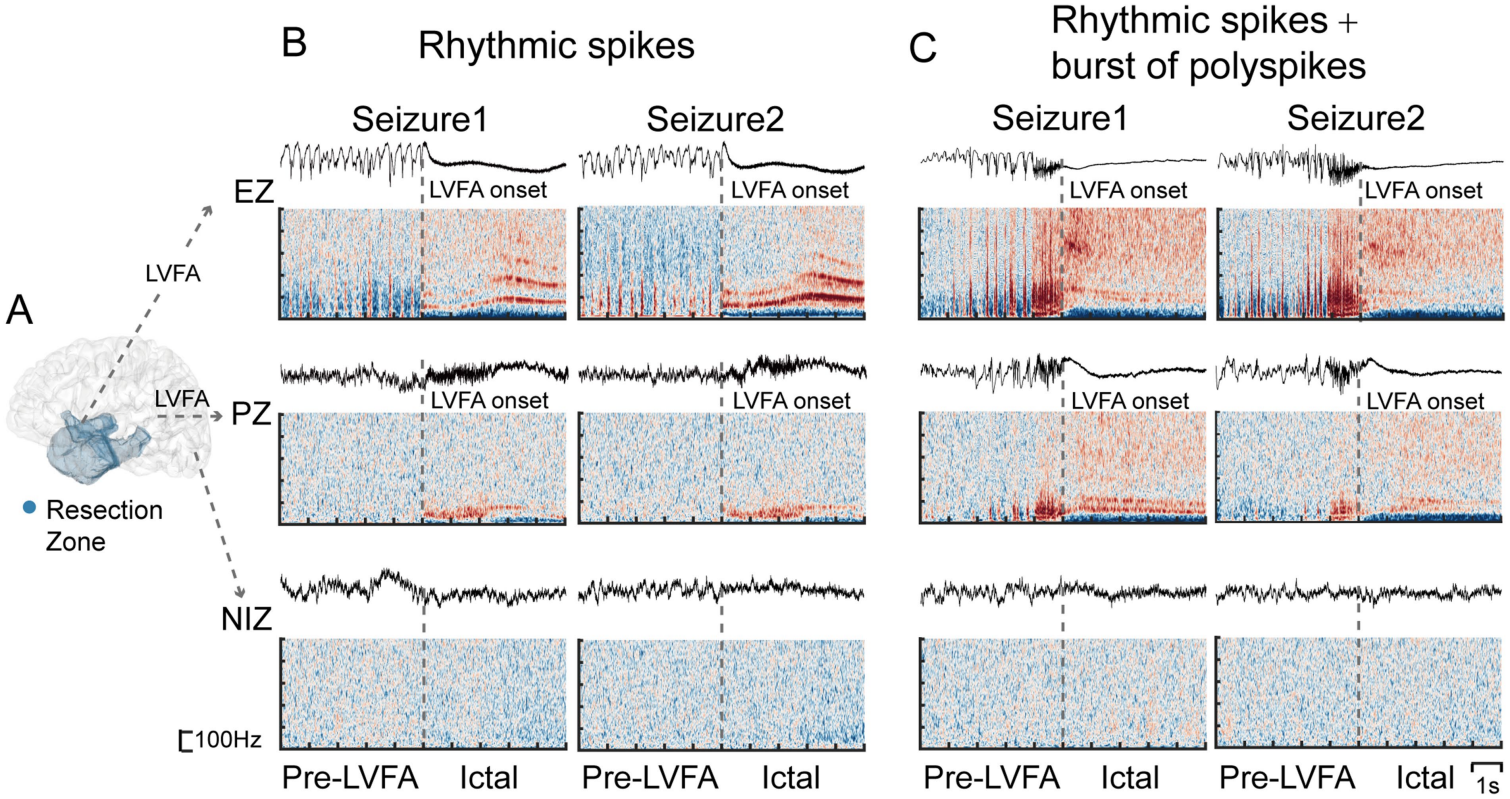
Definition of the EZ, PZ and NIZ along with their corresponding SEEG signals and time-frequency plots during two seizures. **(A)** The definition of the EZ, PZ, and NIZ, with the blue region marking the resection zone. SEEG signals and time-frequency plots from example electrodes in the three regions across two seizures are presented, with the pre-LVFA pattern showing rhythmic spikes **(B)** and rhythmic spikes followed by bursts of polyspikes **(C)**, using a bipolar reference and wavelet transform for time-frequency analysis. Gray dotted lines mark the onset of LVFA.

To examine reproducibility during seizure progression, three 10-s periods were selected: interictal (150–140 s before LVFA onset), pre-LVFA (10 s before LVFA onset), and ictal (first 10 s after LVFA onset).

### 2.3 SEEG analysis

Four LVFA seizures per patient were included. If a patient had more than four seizures, four were randomly selected. Data were referenced using a bipolar montage and analyzed with EEGLAB (21) and the FieldTrip toolbox (22) (both implemented in MATLAB).

### 2.4 Calculating between-seizure reproducibility of waveforms and power spectra

We calculated the reproducibility of raw signal waveforms and periodic power spectra for each electrode within each zone and period. To ensure temporal alignment across seizures, we used a semi-automated algorithm combining FOOOF algorithm (23) and WBS algorithm (24) to detect LVFA onset (see in Appendix and Supplementary Figure 15). In each seizure, the zero point for electrodes within the EZ and PZ was defined by the LVFA onset detected on each electrode, while NIZ electrodes, which lack LVFA, were aligned to the earliest LVFA onset across EZ and PZ to ensure temporal comparability.

After aligning the periods, we employed the nonlinear regression coefficient *h^2^* to assess the reproducibility of raw signal waveforms across seizures within each zone and period. Given the nonlinear nature of SEEG signals, *h^2^* is more suitable for EEG/SEEG analysis than traditional linear methods (25, 26). This approach quantifies the nonlinear statistical dependence between two signals (27). Specifically, the amplitude of signal *Y* is modeled as a nonlinear regression function of the amplitude of signal *X*. The conditional variance, *var*[*y(t)|x(t)*], represents the residual variance of *Y* given *X*. The nonlinear regression coefficient *h^2^*, quantifies the fraction of *Y’s* variance explained by *X* and is computed as:


$$h^{2}=1-\frac{\mathrm{var}[y(t)|x(t)]}{\mathrm{var}[y(t)]}$$

**Figure 2:**
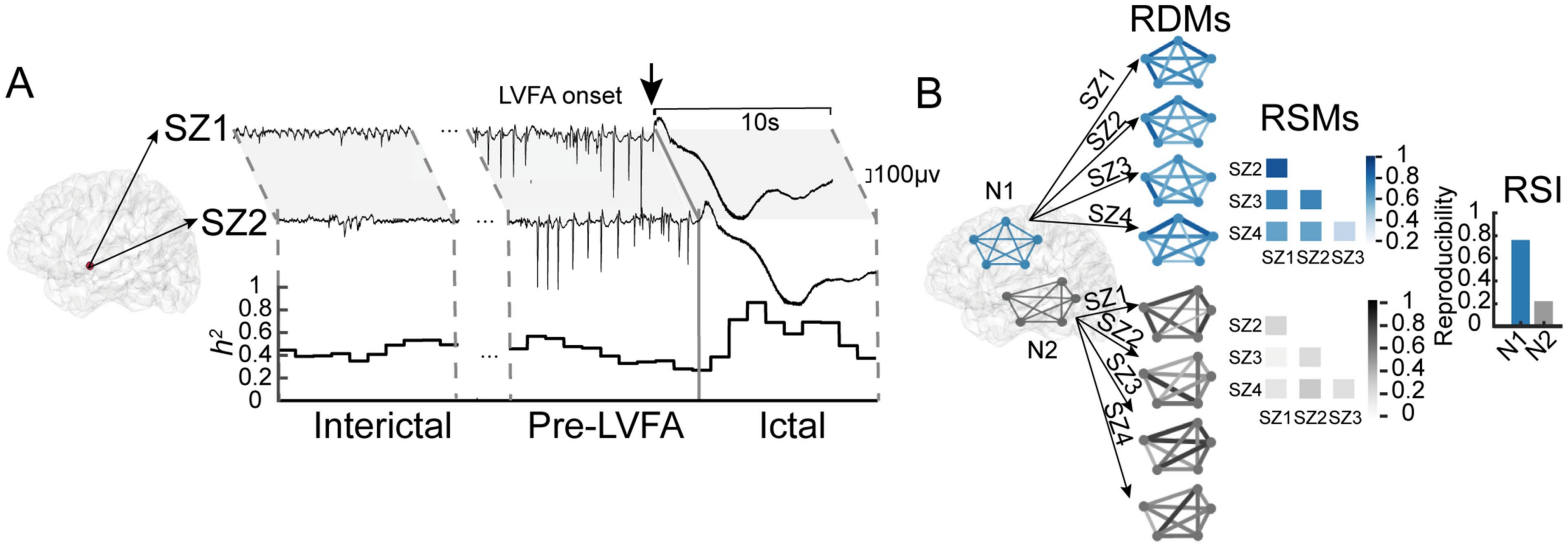
Illustration of calculations for between-seizure reproducibility of raw signals and connectivity patterns. **(A)** Raw SEEG signals from two LVFA seizures (SZ1 and SZ2) recorded from the same electrode in patient 10. Reproducibility of raw signals during interictal, pre-LVFA, and ictal periods was assessed using nonlinear correlation (*h^2^*) with a 2 s sliding window and 1 s step. Line graphs show temporal *h^2^*. **(B)** The computation of between-seizure reproducibility of connectivity using simulated data. Brain diagrams display electrode connections in Network 1 (N1, blue) and Network 2 (N2, black). The RDMs shows connectivity patterns across four seizures, with line thickness and color indicating signal differences—thicker, darker lines denote greater differences. The RSMs quantifies reproducibility across RDMs, with darker colors indicating higher reproducibility. The RSI, calculated from the average RSMs values, is higher for N1, indicating greater between-seizure reproducibility of connectivity patterns in N1.

The value of *h^2^* range from 0 to 1. When *X* and *Y* are completely independent, the conditional variance (*var*[*y(t)|x(t)*]) equals the total variance of *Y*, resulting in *h^2^* = 0. As the dependence increases, the conditional variance decreases, and *h^2^* approaches 1. In this study, *h^2^* quantifies the consistency of instantaneous amplitude fluctuations, revealing whether signals from the same region exhibit stable temporal evolution across seizures. In practice, for each electrode, *h^2^* values for raw SEEG signals from two seizures within each period were calculated using a 2-s sliding window with a 1-s step. Figure 2A illustrated this process. This *h^2^* values were averaged across all seizure pairs and all electrodes within each region, and then averaged for all sliding windows within each period. To account for potential time delays between signals, *h^2^* was calculated across multiple delays, and the maximum value was selected. For asymmetric nonlinear relationships, both *h^2^(xy)* and *h^2^(yx)* were computed, and the higher of the two was used as the representative value. Notably, under linear relationships, *h^2^* is equivalent to the Pearson correlation coefficient.

Reproducibility of periodic power spectra across seizures within each zone and period was calculated using Pearson correlation (*r*), including delta (1–4 Hz), theta (4–8 Hz), alpha (8–13 Hz), beta (13–30 Hz), gamma (30–80 Hz), ripple (80–250 Hz), and fast ripple (250–500 Hz). Pearson correlation is well-suited for the normalized spectral features extracted using the FOOOF algorithm (28), which effectively removes aperiodic background activity. In this study, it was used to quantify the stability of spectral power distributions across seizures within specific frequency bands. In practice, periodic power spectra were extracted using the FOOOF algorithm for each window, excluding non-periodic components. The calculation procedures followed the same approach as for assessing the reproducibility of raw signals.

### 2.5 Calculating between-seizure reproducibility of within- and between-region connectivity patterns

We calculated the reproducibility of connectivity patterns across seizures within and between the EZ, PZ, and NIZ during various periods using representational similarity analysis (RSA). In RSA, neural states are characterized by representational dissimilarity matrices (RDMs), which encode the functional connectivity patterns between regions. The stability of these patterns across time or conditions is quantified using representational similarity matrices (RSMs) (29). In practice, the zero point for all electrodes was determined by the earliest LVFA onset to align periods across seizures and ensure synchronization across electrodes within a seizure. Connections of interest were classified as within-region (NIZ, PZ, EZ) or between-region (NIZ–PZ, NIZ–EZ, PZ–EZ). For each patient, an iterative distance-matching procedure was used to control for confounding effects of electrode pair distance and number. The maximal equal number of electrode pairs across the three connections within each category was iteratively selected, and Euclidean distances were compared using a one-way ANOVA to ensure no significant differences (*p* > 0.05). Only distance-matched sets were retained for subsequent analyses (30). Connectivity patterns were quantified using RDM for each seizure, period, and connection. Dissimilarities were calculated as 1 minus the Pearson correlation of the 10-s raw signals between all selected electrode pairs. Between-seizure reproducibility was assessed using RSA: for each connection and period, Kendall’s τau-a correlations were computed between the RDMs of all seizure pairs, resulting in a seizure-by-seizure RSM (31). The representational similarity index (RSI) was then derived by averaging the off-diagonal lower-triangle values of the RSM. Figure 2B illustrated this analysis process. Finally, connectivity reproducibility was compared separately within- and between- region across patients.

### 2.6 Statistical analysis

For each reproducibility feature, including the nonlinear correlation (*h^2^*) of raw SEEG signals, the Pearson correlation (*r*) of periodic power spectra in each frequency band, and the representational similarity index of connectivity patterns both within- and between- region, the values were averaged for each region and period. A one-way repeated ANOVA and planned paired *t*-tests (without multiple comparison corrections) was conducted to assess differences in reproducibility across regions for each period. *p*-value < 0.05 was considered statistically significant.

## 3 Results

### 3.1 Between-seizure reproducibility of waveforms (raw signals)

**Figure 3:**
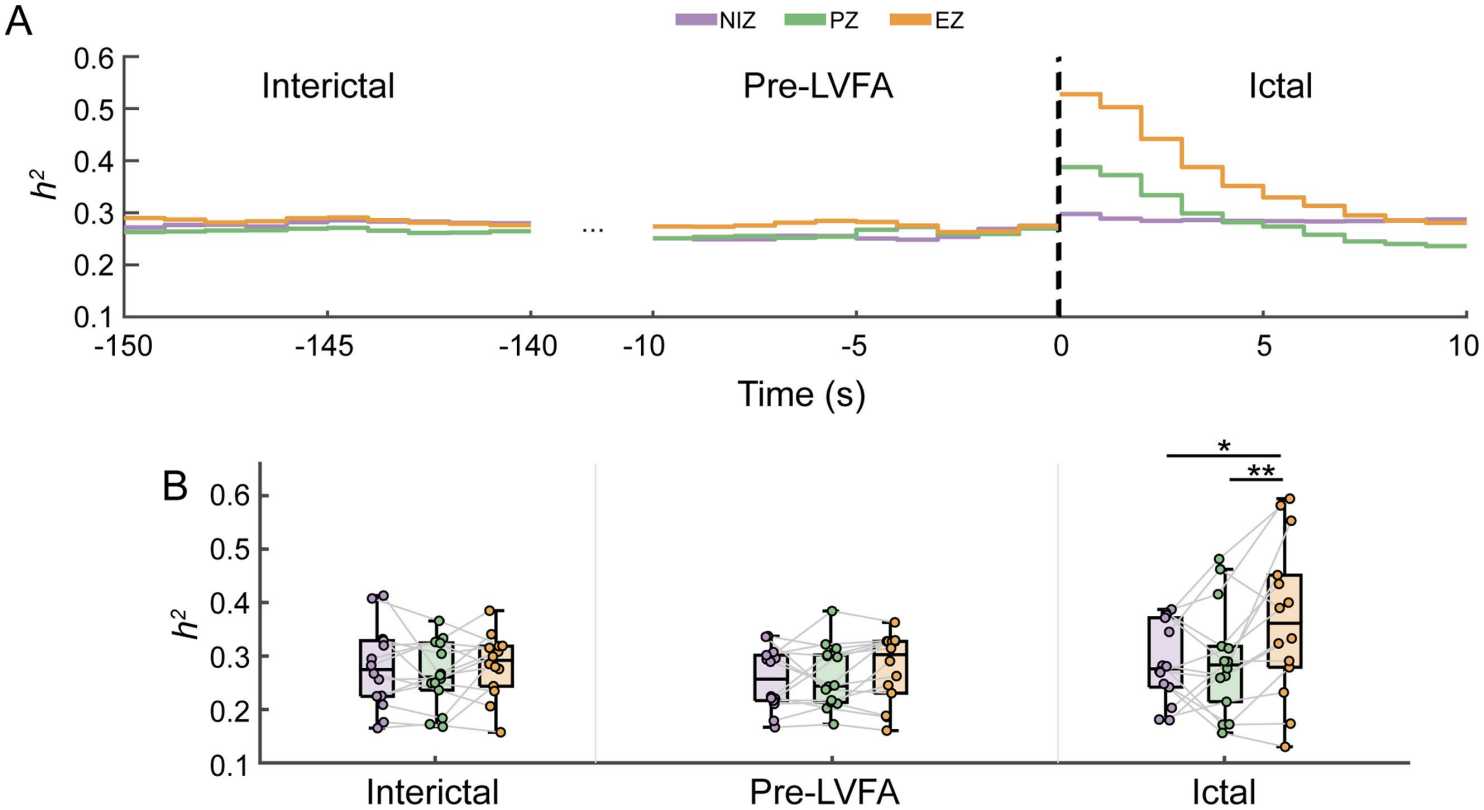
Between-seizure reproducibility of raw signals in NIZ, PZ and EZ during interictal, pre-LVFA, and ictal periods. Time courses of *h^2^* **(A)** in the three phases are shown as a function of the EZ, PZ, and NIZ, with LVFA seizure onset marked at time 0 (dotted vertical lines). **(B)** For each phase, the average *h^2^* values across all windows were calculated, and the mean *h^2^* values for the three regions are presented using boxplots and scatter plots during the interictal, pre-LVFA, and ictal periods. Each dot represents one patient, and lines connect NIZ, PZ, and EZ within the same patient. (**p < 0.01, *p < 0.05).

We assessed the reproducibility of raw SEEG signal waveforms across the EZ, PZ, and NIZ during interictal, pre-LVFA, and ictal periods using *h^2^* estimates to measure the similarity of waveform amplitude variations over time (see Methods). Significant differences in *h^2^* values were observed across the three regions only during the ictal period (*F(2, 26)* = 5.16, *p* = 0.013; Figure 3), not during the interictal (*F(2, 26)* = 0.84, *p* = 0.443; Figure 3B) or pre-LVFA periods (*F(2, 26)* = 1.19, *p* = 0.32; Figure 3B). Further paired t-tests revealed that significantly higher *h^2^* values in the EZ compared to both the PZ (*t(13)* = 3.19, *p* = 0.007) and the NIZ (*t(13)* = 2.18, *p* = 0.048), with no significant difference observed between the PZ and NIZ (*t(13)* = 0.22, *p* = 0.829) (Figure 3B).

### 3.2 Between-seizure reproducibility of power spectra in different frequency bands

**Figure 4:**
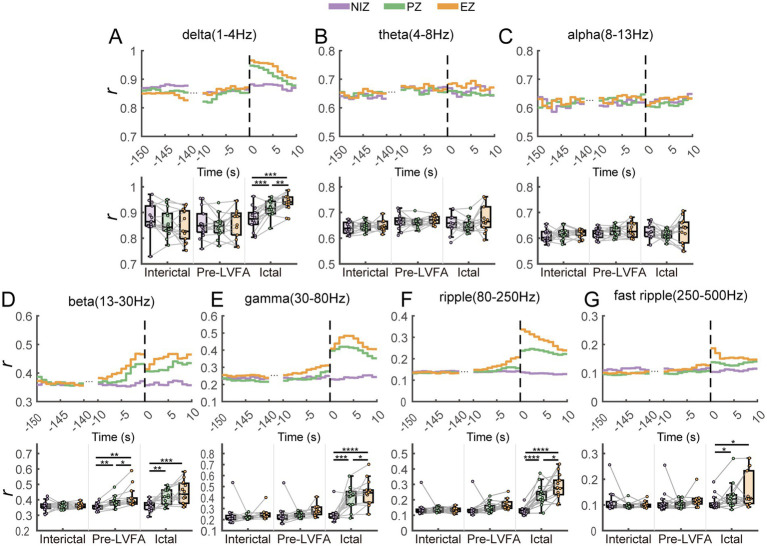
Between-seizure reproducibility of spectra in different frequency bands in NIZ, PZ and EZ during interictal, pre-LVFA, and ictal periods. The frequency bands of interest include the delta **(A)**, theta **(B)**, alpha **(C)**, beta **(D)**, gamma **(E)**, ripple **(F)**, and fast ripple **(G)**. The upper part of each panel shows the time series of power spectral *r* values between different seizures during the interictal, pre-LVFA, and ictal phases as a function of the three regions (EZ, PZ, and NIZ), with LVFA seizure onset marked at time 0 (dotted line). The lower part displays the average *r* values across the whole time windows for each phase as a function of the three regions. (****p < 0.0001, ***p < 0.001, **p < 0.01, *p < 0.05).

To further investigate the reproducibility of frequency characteristics in periodic power spectra across seizures, we further calculated the *r* values of periodic oscillatory activity for each frequency band of interest in the three regions during the interictal, pre-LVFA, and ictal periods, specifically representing the similarity in power intensity within specific frequency ranges (see methods). During the interictal period, no significant differences in *r* values were found across regions for all frequency bands (all *p >* 0.05; Figures 4A–G). In the pre-LVFA period, significant between-regional differences in *r* values were observed only in the beta band (*F(2, 26)* = 11.47, *p* = 0.002; Figure 4D), and the EZ had significantly higher *r* value than both PZ (*t(13)* = 2.32*, p* = 0.037) and NIZ (*t(13)* = 3.73, *p* = 0.003), while the PZ had higher *r* value than NIZ (*t(13)* = 3.96, *p* = 0.002) (Figure 4D). During the ictal period, significant between-regional differences in *r* values were found in the delta (*F(2, 26)* = 20.8, *p <* 0.0001), beta (*F(2, 26) =* 12.76, *p* = 0.0001), gamma (*F(2, 26)* = 27.27, *p <* 0.0001), ripple (*F(2, 26)* = 34.77, *p* < 0.0001), and fast ripple (*F(2,26)* = 5.17, *p* = 0.013) bands (Figures 4A,D–G). The *r* value in EZ (delta: *t(13)* = 5.04*, p =* 0.0002; beta: *t(13)* = 4.43*, p =* 0.0007; gamma: *t(13)* = 6.05*, p <* 0.0001; ripple: *t(13)* = 7.79, *p* < 0.0001; fast ripple: *t(13)* = 2.77*, p =* 0.016) and PZ (delta: *t(13)* = 4.06*, p =* 0.001; beta: *t(13)* = 3.9, *p =* 0.002; gamma: *t(13)* = 5.13, *p* = 0.0002; ripple: *t(13)* = 6.05, *p* < 0.0001; fast ripple: *t(13)* = 2.27, *p* = 0.041) were both significantly higher than those in NIZ for these bands (Figures 4A,D–G). More importantly, *r* values in EZ were higher than those in PZ for delta, gamma, and ripple bands (delta: *t(13)* = 3.65, *p* = 0.003; gamma: *t(13)* = 2.24, *p* = 0.043; ripple: *t(13)* = 2.7, *p* = 0.018) (Figures 4A,E,F).

### 3.3 Between-seizure reproducibility of within- and between-region connectivity patterns

**Figure 5:**
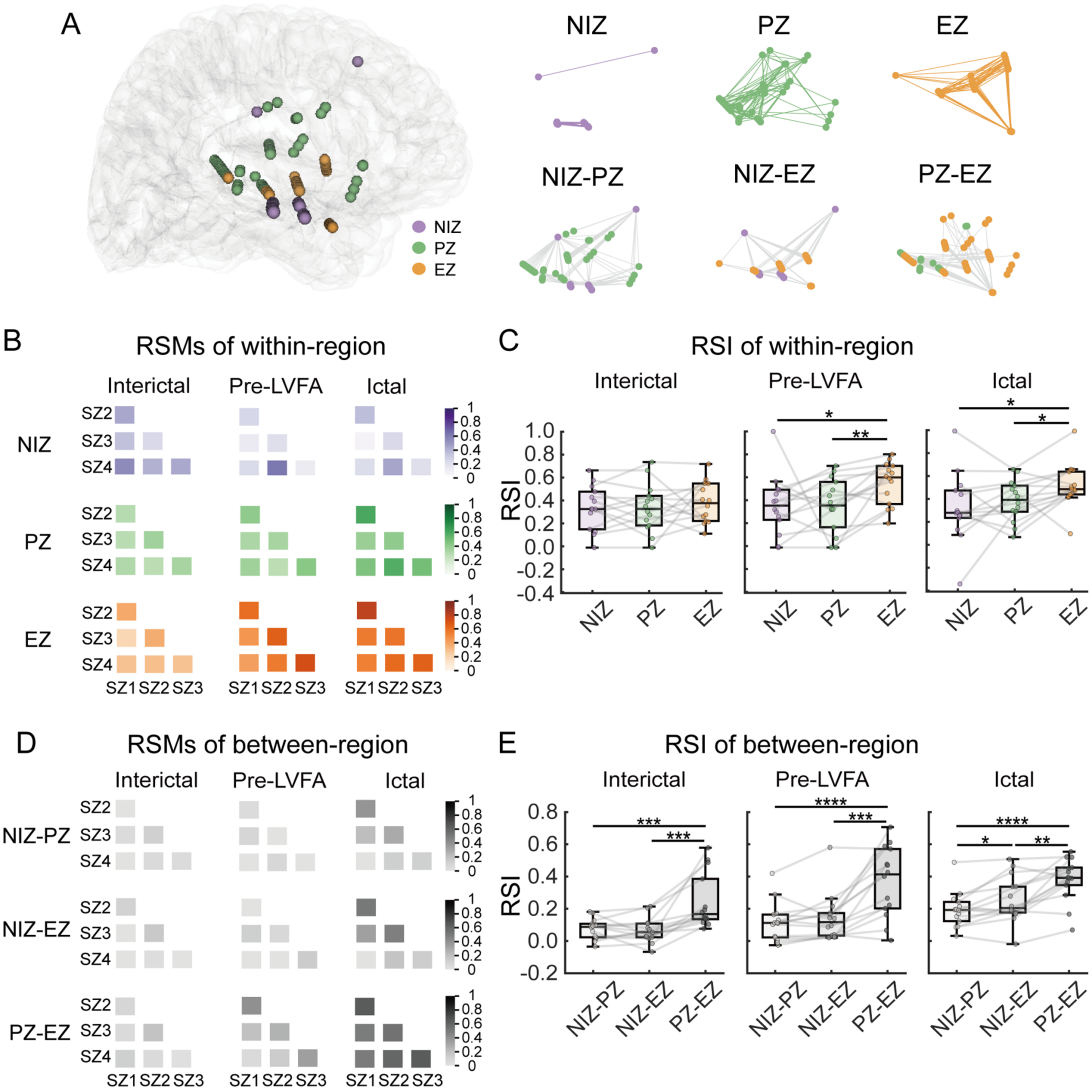
Between-seizure reproducibility of connectivity patterns within- and between-region during interictal, pre-LVFA, and ictal periods. **(A)** Electrode distribution across zones in patient 10, with within- and between-region connections are illustrated. The lines represent the connection relationships. **(B)** RSMs for within-region connectivity during the interictal, pre-LVFA, and ictal periods. Each matrix represents six seizure pairs from four seizures, with color depth indicating reproducibility (darker colors = higher similarity). **(C)** RSI for within-region connectivity, averaged across RSMs, during interictal, pre-LVFA, and ictal periods. **(D)** RSMs for between-region connectivity during the same periods. **(E)** RSI for between-region connectivity during the same periods. (****p < 0.0001, ***p < 0.001, **p < 0.01, *p < *0.05*).

To further test the between-seizure reproducibility of epileptic network dynamics, we examined the reproducibility of both within- and between-region connectivity patterns during the interictal, pre-LVFA, and ictal periods by RSI (see methods). Figure 5A illustrated an example of electrode distribution across regions in a single patient, showing both within- and between-regions connections. For the within-region connectivity, significant differences in RSI values were observed across the three regions during the pre-LVFA (*F(2, 26)* = 4.05, *p* = 0.029) and ictal (*F(2, 26)* = 4.16, *p* = 0.027) periods, but not during the interictal (*F(2, 26)* = 0.41, *p* = 0.666) period (Figure 5C). During the pre-LVFA and ictal periods, the RSI within the EZ exhibited significantly higher reproducibility across seizures than within the PZ (pre-LVFA: *t(13)* = 3.53, *p* = 0.004; ictal: *t(13)* = 2.41, *p* = 0.032) and NIZ (pre-LVFA: *t(13)* = 2.33, *p* = 0.037; ictal: *t(13)* = 2.58, *p* = 0.023), with no significant difference was found between PZ and NIZ (pre-LVFA: *t(2, 26)* = 0.03, *p* = 0.978; ictal: *t(13)* = 0.83, *p* = 0.422) (Figure 5C). For the between-region connectivity, the representational similarity matrices during the interictal, pre-LVFA, and ictal periods in this patient was shown in Figure 5D. Significant differences in RSI values were observed across these regions during all periods (interictal: *F(2, 26)* = 18.6, *p* = 0.0002; pre-LVFA: *F(2, 26)* = 25.27 *p <* 0.0001; ictal: *F(2, 26)* = 24.67, *p <* 0.0001) (Figure 5E). The RSI of EZ-PZ exhibited significantly higher than the NIZ-PZ (interictal: *t(2, 26)* = 4.57*, p* = 0.0005; pre-LVFA: *t(13)* = 5.97 *p <* 0.0001; ictal: *t(2, 26)* = 7.06*, p <* 0.0001) and NIZ-EZ (interictal: *t(13)* = 4.53, *p =* 0.0006; pre-LVFA: *t(13)* = 4.63 *p =* 0.0005; ictal: *t(13)* = 3.89, *p* = 0.002) during all periods (Figure 5E). The RSI of NIZ-EZ connectivity was more significantly higher than NIZ-PZ only during ictal period (*t(13)* = 2.86, *p* = 0.014) (Figure 5E).

**Figure 6:**
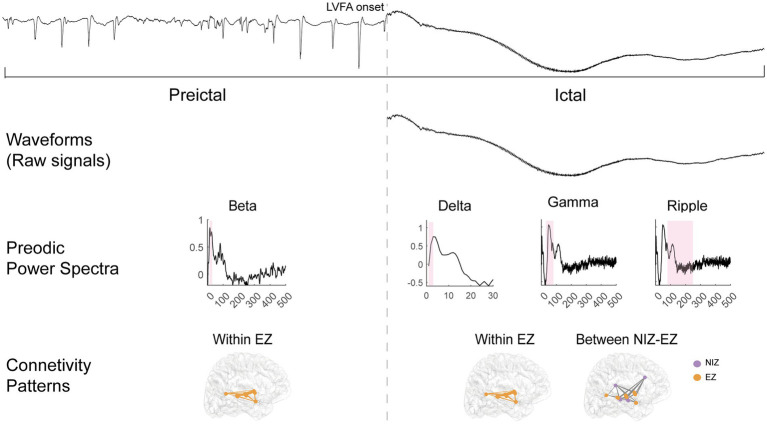
Reproducible features could differentiate EZ from PZ. The first row shows an example of an LVFA seizure from the pre-LVFA to ictal periods, with the LVFA seizure onset marked by a dotted line. The second to fourth rows display the following reproducible features that differentiate the EZ from the PZ: ictal waveforms; pre-LVFA beta and ictal delta, gamma, and ripple power spectra (with the pink bar corresponding to the current frequency band); and pre-LVFA connectivity patterns within the EZ, along with ictal connectivity patterns both within the EZ and between the NIZ and EZ.

Taken together, the temporally evolving reproducible features distinguishing the EZ from the PZ are summarized in Figure 6. Robustness was further validated by shifting the interictal baseline window (−140 to −130 s; −130 to −120 s), which yielded consistent results. In addition, all planned pairwise comparisons were reanalyzed with FDR correction, and all key contrasts between EZ and PZ remained significant (see in Supplementary Tables 1, 2). We also analyzed one patient who underwent a failed first resection followed by a successful second resection. The patient’s clinical information, analytic methods, and results are provided in Appendix 1: Supplementary Case Analysis. Consistent with the main cohort, this case demonstrated greater reproducibility of both waveforms and connectivity in EZ compared with PZ across seizures. Detailed statistical results are available in the Supplementary Figures 17–23.

## 4 Discussion

Previous studies had demonstrated reproducibility in waveform patterns (13, 14), frequency range (6), and connectivity patterns (17) across seizures. However, the temporal dynamics of these reproducible features throughout the seizure, as well as their ability to distinguish the EZ from the PZ and NIZ, remain poorly understood. To address this, we compared between-seizure reproducibility of raw signals, power spectra, and connectivity patterns across the EZ, PZ, and NIZ, across the interictal, pre-LVFA, and ictal periods.

### 4.1 Higher SEEG waveforms reproducibility in the EZ specifically during the ictal period

Our findings demonstrated that ictal waveforms reproducibility across seizures was significantly higher in the EZ compared to other regions (Figure 3B), despite the presence of LVFA in the PZ. Waveform shapes provide valuable insights into the state of oscillatory generators in the brain (32). The reproducibility of waveform shapes within the EZ across seizures may indicate stereotypically neuronal activity in these generators.

The simultaneous increase of inhibitory interneuron firing rates and suppression of principal neuron activity has been shown to occur during LVFA in humans, preceding the subsequent increase in principal neuron activity (33). Microelectrode studies supported the reproducibility of ictal firing patterns of individual neurons during LVFA seizures (34, 35). Moreover, spatiotemporally organized neuronal firing patterns exhibited high consistency and stereotypy across seizures (36, 37). Schevon et al. further revealed that neuronal firing patterns in the presumed ictal penumbra exhibited limited reproducibility across seizures, whereas the core recruited area where ictal discharges originate, showed high reproducibility (12). Together with previous evidence, higher reproducibility of ictal SEEG waveforms in the EZ may reflect spatially and temporally stereotyped neuronal activity at the same site, including both inhibitory interneurons and principal neurons. These results further emphasize the value of waveforms reproducibility as a biomarker for distinguishing the EZ from PZ during the ictal period.

### 4.2 Higher spectral reproducibility in the EZ

Our results showed that ictal power spectra reproducibility both in gamma (Figure 4E) and ripple (Figure 4F) bands of high frequencies, and in delta band (Figure 4A) of low frequencies across seizures is significantly higher in the EZ than in the PZ. The higher reproducibility of high frequencies results was consistent with previous findings (6) and further suggested that power spectra of both fast and slow activity in the EZ remains stable across seizures. Different frequency bands are generally believed to reflect the activity of specific neuronal populations (38). During seizure onsets, the high frequency bands have been associated with fast inhibitory interneuron activity, and the low-frequency bands have been associated with the suppression of low-frequency activity due to the inhibition of slow pyramidal neuron discharges by the fast inhibitory interneuron activity (10, 39). Thus, these results may indicate the stability of activity within these different neuronal populations in the EZ across seizures. In contrast to the ripple activity, the fast ripple power spectra did not exhibit higher reproducibility in the EZ than PZ (Figure 4G), which may be attributed to the higher occurrence rate of ripples compared to fast ripples during LVFA (40).

Moreover, during the pre-LVFA period, reproducibility of power spectra in the beta band was already higher in the EZ than PZ (Figure 4D). The majority of pre-ictal patterns in this study involve rhythmic spikes (Figures 1B,C, Table 1). A recent model proposed that increased pyramidal cell activity during the interictal phase triggers distant inhibitory interneuron activity, resulting in rhythmic spikes during the pre-LVFA period (41). Therefore, the pre-LVFA higher reproducibility of beta-band power spectra within EZ may be associated with the pre-LVFA rhythmic spikes which likely reflects the stereotyped activity of intermediate inhibitory neurons. Taken together, the present findings underscore the potential of assessing pre-LVFA beta, and ictal fast and slow activity power spectra reproducibility across multiple seizures as an important criterion for EZ localization.

### 4.3 Higher connectivity reproducibility in the EZ

Our results showed that for within-region connectivity, significantly higher reproducibility of functional connectivity patterns was found in the EZ than in PZ during both the pre-LVFA and ictal periods (Figure 5C). A study proposes that simultaneous LVFA observed within the EZ is not driven by a propagation mechanism, but rather by similar pathological changes within the EZ (41). The organization of the EZ is shaped by these changes, which lead to the cascading of synaptic connection nodes between different regions through the same mechanism, requiring connectivity between remote neuronal populations (41). These findings further support the concept of the EZ as a self-organizing network (16), and our results further confirm that this self-organizing pattern remains reproducible across seizures. In contrast, the PZ showed no significantly higher reproducibility of within-region connectivity than the NIZ (Figure 5C), implying that the PZ activity patterns might be driven primarily by its connectivity with the EZ (42).

Accordingly, for the between-region connectivity, we found that the reproducibility of connectivity patterns of PZ-EZ was significantly higher than that of NIZ-PZ and NIZ-EZ throughout the interictal, pre-LVFA, and ictal periods (Figure 5E), reflecting highly reproducible patterns of functional coupling within the epileptic network across both periods and seizures. A previous study suggested that even during the interictal period, the EZ and PZ exhibit preferential coupling (43). This stereotyped preferential coupling between the EZ and PZ may facilitate the propagation of epileptic activity. Targeting or disrupting this stable pathway could attenuate seizure spread and represent a potential therapeutic strategy. Interestingly, even the connectivity between the EZ and NIZ became more reproducible than that between the PZ and NIZ during the ictal period (Figure 5E), suggesting that EZ involvement during seizures contributes to a robust and reproducible network coupling, including connections between the EZ and NIZ. Therefore, these results support the notion that epilepsy is a self-organized phenomenon originating from a small, localized EZ, and the EZ drives seizure initiation and propagation.

## Data Availability

The original contributions presented in the study are included in the article/Supplementary material, further inquiries can be directed to the corresponding authors.
