# Supplementary material for: Reproducibility of temporally evolving seizure patterns and network connectivity in focal epilepsy
Source: Front Neurol. 2025 Nov 11;16:1617317. doi: 10.3389/fneur.2025.1617317 (PMC12644071; doi:10.3389/fneur.2025.1617317)
Supplement: Supplementary file 1 [file Table_1.docx]

Appendices

# Supplementary Case Analysis

­­This male patient developed epilepsy at age 10 years. MRI was negative and histopathology was non-specific, whereas PET demonstrated bilateral insular and opercular hypometabolism. At age 27, a first resection (posterior insula and central operculum) was performed, but seizures persisted (Engel III). At age 31, a second resection involving the anterior/mid-cingulate cortex (ACC/MCC) and Brodmann areas 8/9 achieved seizure freedom (Engel I). Resection areas are illustrated in Supplementary Figure 16A.

The reproducibility framework applied in the main analyses was adopted, encompassing three temporal windows (interictal, pre-LVFA, ictal) and three reproducibility measures (waveform, spectral power, connectivity). Five seizures with LVFA onset were recorded, of which four were analyzed. Electrode contacts were categorized as NIZ (n = 36), PZ (n = 44), EZ1 (n = 9; first resection), and EZ2 (n = 13; second resection) (Supplementary Figure 16B). As variance-based group statistics were not applicable to a single subject, statistical significance was evaluated using permutation testing (1,000 iterations), with effects deemed significant when exceeding the 95th percentile of the null distribution.

(i) Waveform and spectral reproducibility: EZ > PZ reproducibility was assessed in the ictal waveform, pre-LVFA beta, ictal delta, ictal gamma, and ictal ripple bands, which had shown significant group-level effects. For each permutation, electrode labels were randomly reassigned, and independent-sample t statistics were recomputed to generate a null distribution.

(ii) Connectivity reproducibility: Within-region (EZ > PZ during pre-LVFA and ictal periods) and between-region contrasts (PZ–EZ vs. NIZ–PZ during interictal, pre-LVFA, and ictal periods; NIZ–EZ vs. NIZ–PZ during ictal period) were examined. For each permutation, connectivity patterns were randomly shuffled, Kendall’s tau-a coefficients were recalculated, and mean differences were compared against the null distribution.

For waveform reproducibility, both EZ1 and EZ2 exhibited significantly higher h² values than PZ during the ictal onset phase (*p < 0.05*, Supplementary Figure 17A). For within-region connectivity, RSI in EZ1 and EZ2 was significantly greater than in PZ during the pre-LVFA (both *p = 0*, Supplementary Figure 18B) and ictal (both *p = 0*, Supplementary Figure 19B) periods. Regarding between-region connectivity, EZ1–EZ2 coupling showed significantly higher RSI than their respective connections with PZ during the pre-LVFA (PZ-EZ1 vs EZ1-EZ2: *p = 0.006*, PZ-EZ2 vs EZ1-EZ2: *p = 0*, Supplementary Figure 21C) and ictal periods (both *p = 0*, Supplementary Figure 23). For PZ-related connectivity, during the interictal period, RSI of PZ–EZ1 was significantly higher than that of PZ–NIZ (*p = 0.037*, Supplementary Figure 20B), whereas PZ–EZ2 did not differ significantly from PZ–NIZ (*p = 0.138*, Supplementary Figure 20B). In contrast, reproducibility of both PZ–EZ1 and PZ–EZ2 was significantly greater than that of PZ–NIZ during both the pre-LVFA (both *p = 0*, Supplementary Figure 21B) and ictal (both *p = 0*, Supplementary Figure 22B) periods. Notably, RSI of PZ–EZ1 was significantly higher than PZ–EZ2 in the pre-LVFA period (*p = 0*, Supplementary Figure 21B), whereas during the ictal period, the RSI of PZ–EZ2 significantly surpassed that of PZ–EZ1 (*p = 0*, Supplementary Figure 22B). For NIZ-related connectivity, during the ictal period, only NIZ–EZ2 reproducibility was significantly higher than NIZ–PZ (*p = 0.047*), while NIZ–EZ1 did not differ significantly from NIZ–PZ (*p = 0.705*) (Supplementary Figure 23C).

# Detecting Seizure Onset at Each Electrode Exhibiting LVFA

We defined the seizure onset as the time point when LVFA occurs, marked by the sudden increase in power within the LVFA frequency band. To detect seizure onset at each electrode exhibiting LVFA, we developed a semi-automated program that follows a three-stage process: (1) identifying the LVFA frequency band for each electrode, (2) calculating the temporal power variations within the identified band, and (3) applying an optimized algorithm to detect the sudden power increase, thereby pinpointing the seizure onset. The specific details of each stage are described below:

## Step 1: determining the LVFA frequency band for each electrode

To identify the specific frequency band of LVFA for each electrode, we employed the fitting oscillations & one over f (FOOOF) algorithm (1). This method models the power spectrum by parameterizing both aperiodic and periodic components. The aperiodic components, which exhibits 1/f noise-like characteristics, were modeled using Lorentzian functions, while the periodic components, including the neuronal rhythmic activities such as LVFA oscillations, were modeled by Gaussian distributions.

Specifically, we first extracted the SEEG data $x[n]$ from an individual electrode, encompassing 20s before and after the seizure onset identified by an expert clinician for each seizure. Here, $n=k\times\Delta T, k=0, 1, \ldots, N$, and $\Delta T$ represents the sampling period. Next, the time-frequency results $TF[w]$ of $x[n]$ were estimated using complex Morlet Wavelet Transform (MWT), evaluating 200 frequencies linearly spaced from 3 to 500 Hz. The Morlet time-frequency index increased linearly from 3 to 50 cycles as the frequency increased.

We then identified the LVFA segment $x_{LVFA}\left[ i \right]$ within $x[n]$ by visually inspecting the SEEG data $x[n]$ and the corresponding time-frequency results $TF[w]$. Finally, the FOOOF algorithm, implemented in Matlab and Python, was used to model the periodic components of $x_{LVFA}\left[ i \right]$, identifying the LVFA frequency band $[f_{1},f_{2}]$ based on the parameters of periodic spectrum. When LVFA manifested as multiple narrow bands (2), we selected the earliest LVFA frequency band observed in the time-frequency signal $TF[w]$.

## Step 2: calculating the time-varying power of the LVFA frequency band

To obtain the temporal power changes within the LVFA frequency band $[f_{1},f_{2}]$, we first performed band-pass filtering on the signal $x[n]$ in the identified LVFA band by applying a third-order Finite Impulse Response (FIR) filter with zero phase distortion. The filtered signals were then processed using a Hilbert transform to compute the time-varying energy $E\left[ n \right]$ for this band (3).

## Step 3: detecting the point of sudden increase in time-varying power

The ictal onset was defined as the time point when the power $E\left[ n \right]$ within the LVFA frequency band $[f_{1},f_{2}]$exhibited a sudden increase. If sole changepoint in power is detected, overly stringent criteria may miss the true onset, while overly relaxed criteria could misidentify unrelated changes as the onset. To address this, we employed the Wild Binary Segmentation (WBS) algorithm from the WBS package in R, which detects multiple changepoints within a time series (4). This method identified multiple statistically significant changes in the power series $E\left[ n \right]$. Among these, the precise seizure onset was determined based on its alignment with both the initial appearance of the LVFA pattern in the raw SEEG signals and in the time-frequency results. This identification was achieved through visual inspection of the raw SEEG signals $x[n]$, the time-frequency analysis results $TF[w]$, and power changes $E\left[ n \right]$. Supplementary Figure 1 illustrated the whole process for identifying the ictal onset for individual electrode.

# References

1. Donoghue T, Haller M, Peterson EJ, Varma P, Sebastian P, Gao R, Noto T, Lara AH, Wallis JD, Knight RT, et al. Parameterizing neural power spectra into periodic and aperiodic components. *Nat Neurosci* (2020) 23:1655–1665. doi: 10.1038/s41593-020-00744-x

2. Grinenko O, Li J, Mosher JC, Wang IZ, Bulacio JC, Gonzalez-Martinez J, Nair D, Najm I, Leahy RM, Chauvel P. A fingerprint of the epileptogenic zone in human epilepsies. *Brain* (2018) 141:117–131. doi: 10.1093/brain/awx306

3. Marple L. Computing the discrete-time “analytic” signal via FFT. *IEEE Transactions on Signal Processing* (1999) 47:2600–2603. doi: 10.1109/78.782222

4. Fryzlewicz P. Wild binary segmentation for multiple change-point detection. *Ann Statist* (2014) 42: doi: 10.1214/14-AOS1245
