# Supplementary material for: Reproducibility of temporally evolving seizure patterns and network connectivity in focal epilepsy
Source: Front Neurol. 2025 Nov 11;16:1617317. doi: 10.3389/fneur.2025.1617317 (PMC12644071; doi:10.3389/fneur.2025.1617317)
Supplement: Supplementary file 2 [file Table_2.docx]

Supplementary Material

# Supplementary Figures and Tables

## Supplementary Table

**Supplementary Table 1.** Paired t-test p value for waveforms, spectra, within-region connectivity patterns.

| Features | NIZ vs PZ | NIZ vs EZ | PZ vs EZ | Effect sizes |
| --- | --- | --- | --- | --- |
| Waveforms (ictal) | 0.829 (0.829) | **0.048 (0.073)** | 0.007 (0.021) | 0.11 |
| Delta spectra (ictal) | 0.001 (0.002) | 0.000226 (0.000678) | 0.003 (0.003) | 0.351 |
| Beta spectra (pre-LVFA) | 0.002 (0.004) | 0.003 (0.004) | 0.037 (0.037) | 0.226 |
| Gamma spectra (ictal) | 0.000192 (0.000288) | 0.0000408 (0.000122) | 0.043 (0.043) | 0.481 |
| Ripple spectra (ictal) | 0.0000408 (0.0000612) | 0.00000298 (0.00000894) | 0.018 (0.018) | 0.557 |
| Within-region connectivity (pre-LVFA) | 0.978 (0.978) | **0.037 (0.055)** | 0.004 (0.011) | 0.146 |
| Within-region connectivity (ictal) | 0.422 (0.422) | 0.023 (0.047) | 0.032 (0.047) | 0.113 |

Parentheses show FDR-adjusted p-values; bold indicates significance changes.

**Supplementary Table 2.** Paired t-test p value for between-region connectivity patterns.

| Features | NIZ-PZ vs NIZ-EZ | NIZ-PZ vs PZ-EZ | NIZ-EZ vs PZ-EZ | Effect sizes |
| --- | --- | --- | --- | --- |
| Between-region connectivity (ictal) | 0.014 (0.014) | 0.00000858 (0.0000257) | 0.002 (0.003) | 0.275 |

Parentheses show FDR-adjusted p-values.

## Supplementary Figures


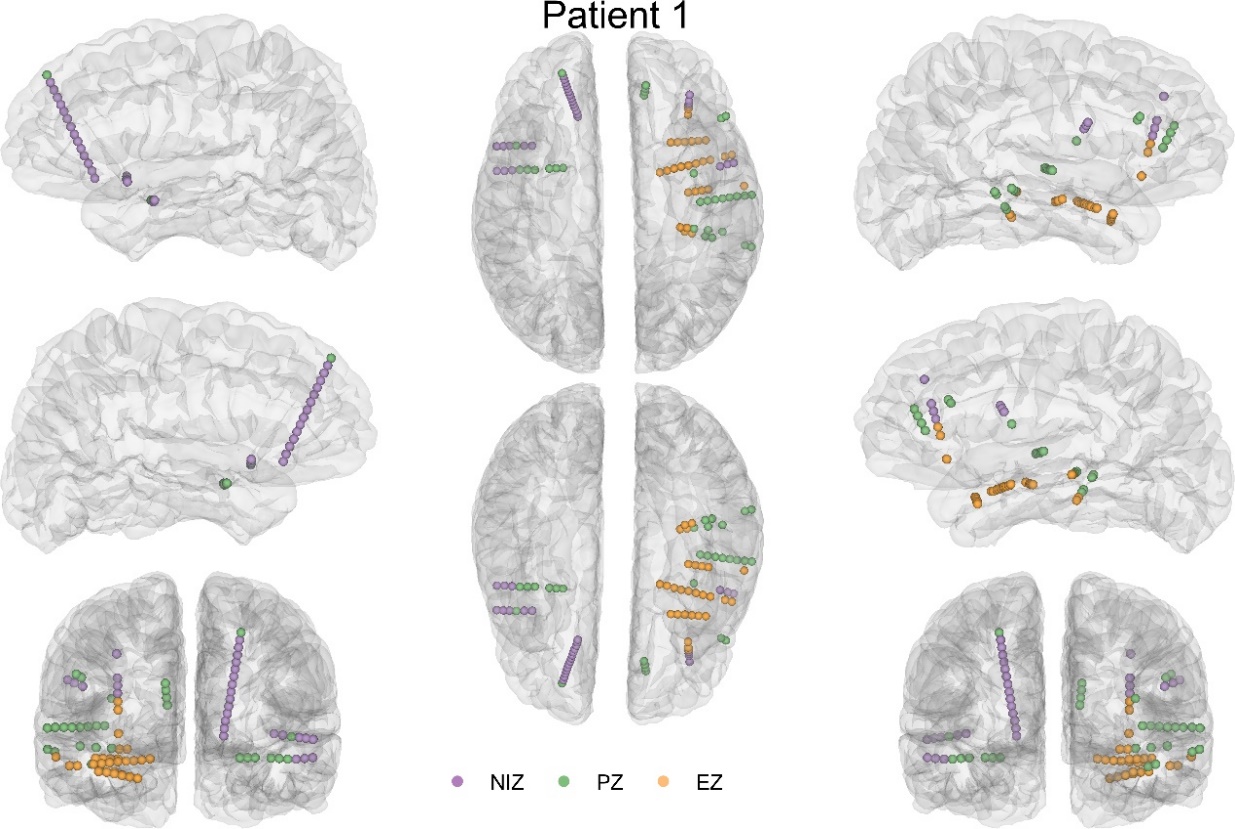


**Supplementary Figure 1.** **Electrode distributions of patient 1.**


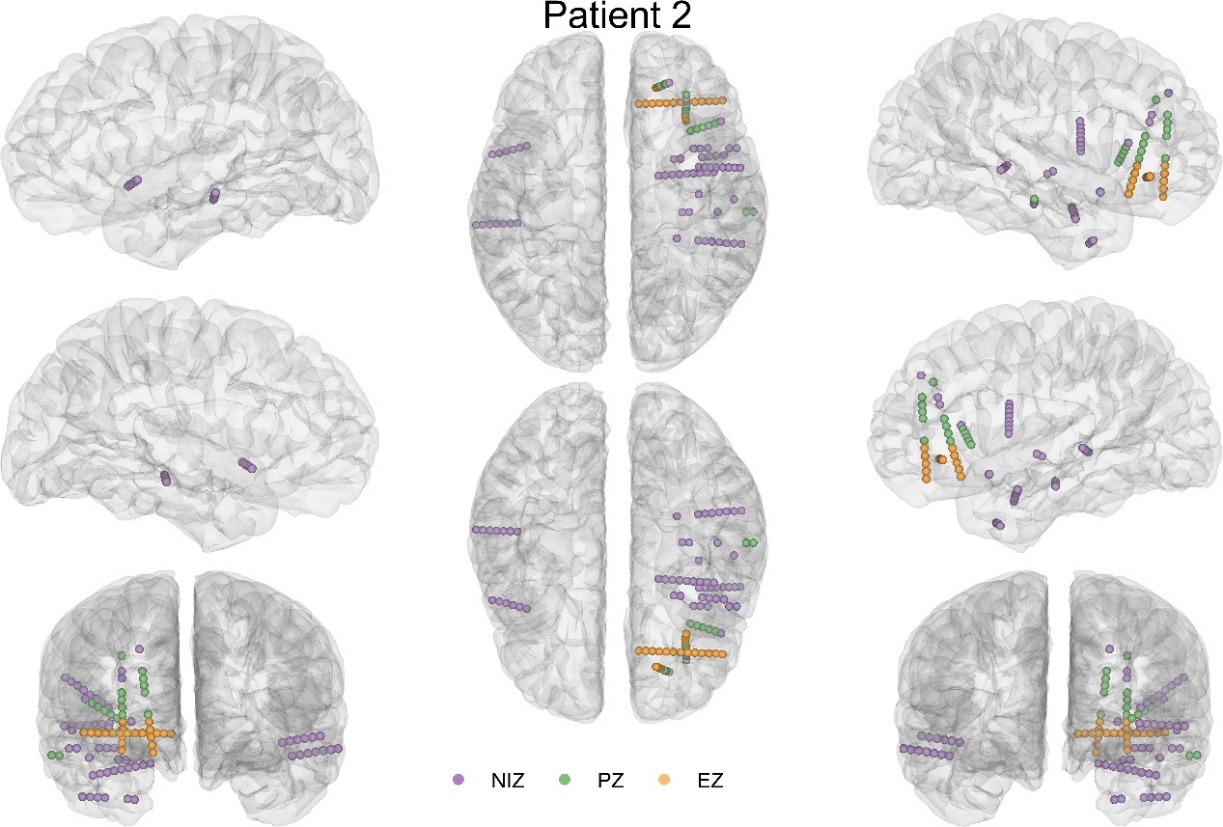


**Supplementary Figure 2.** **Electrode distributions of patient 2.**


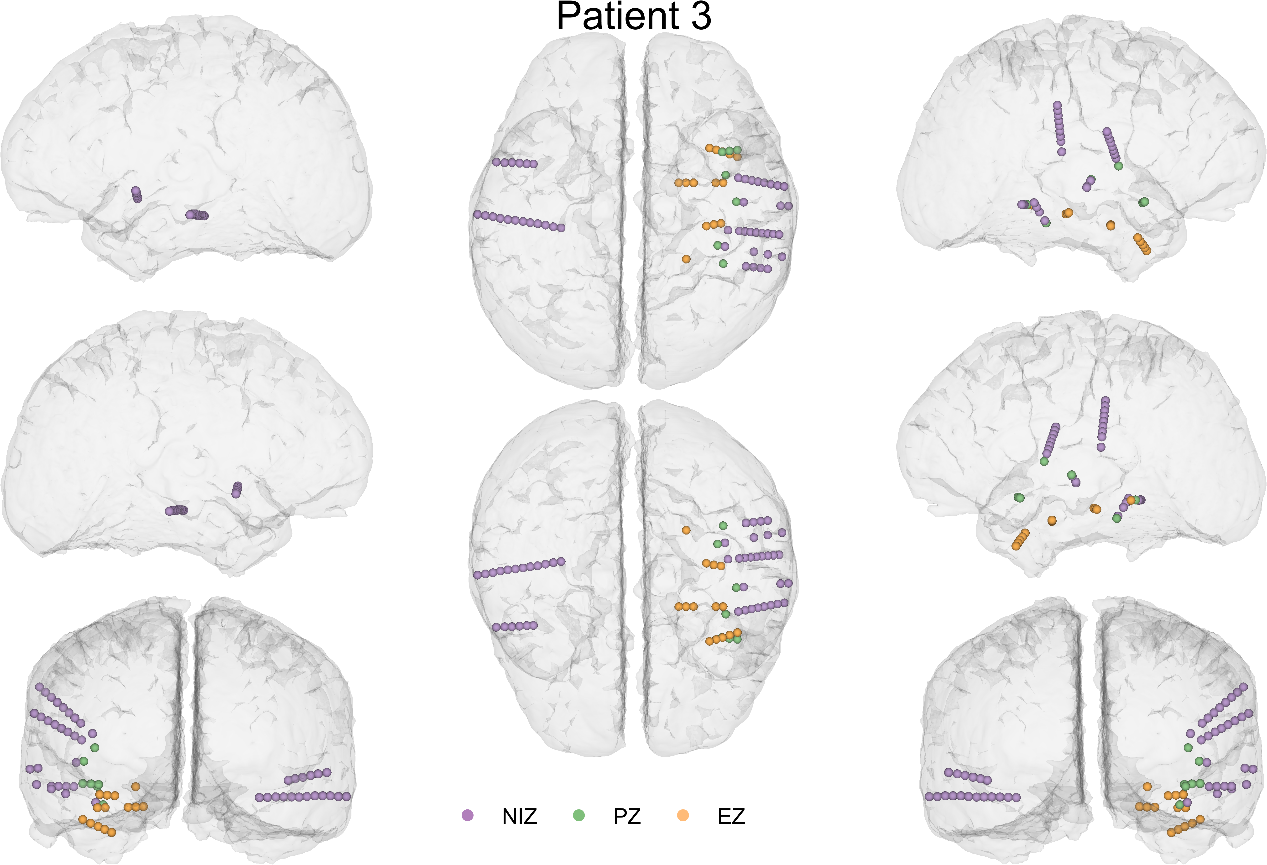


**Supplementary Figure 3.** **Electrode distributions of patient 3.**


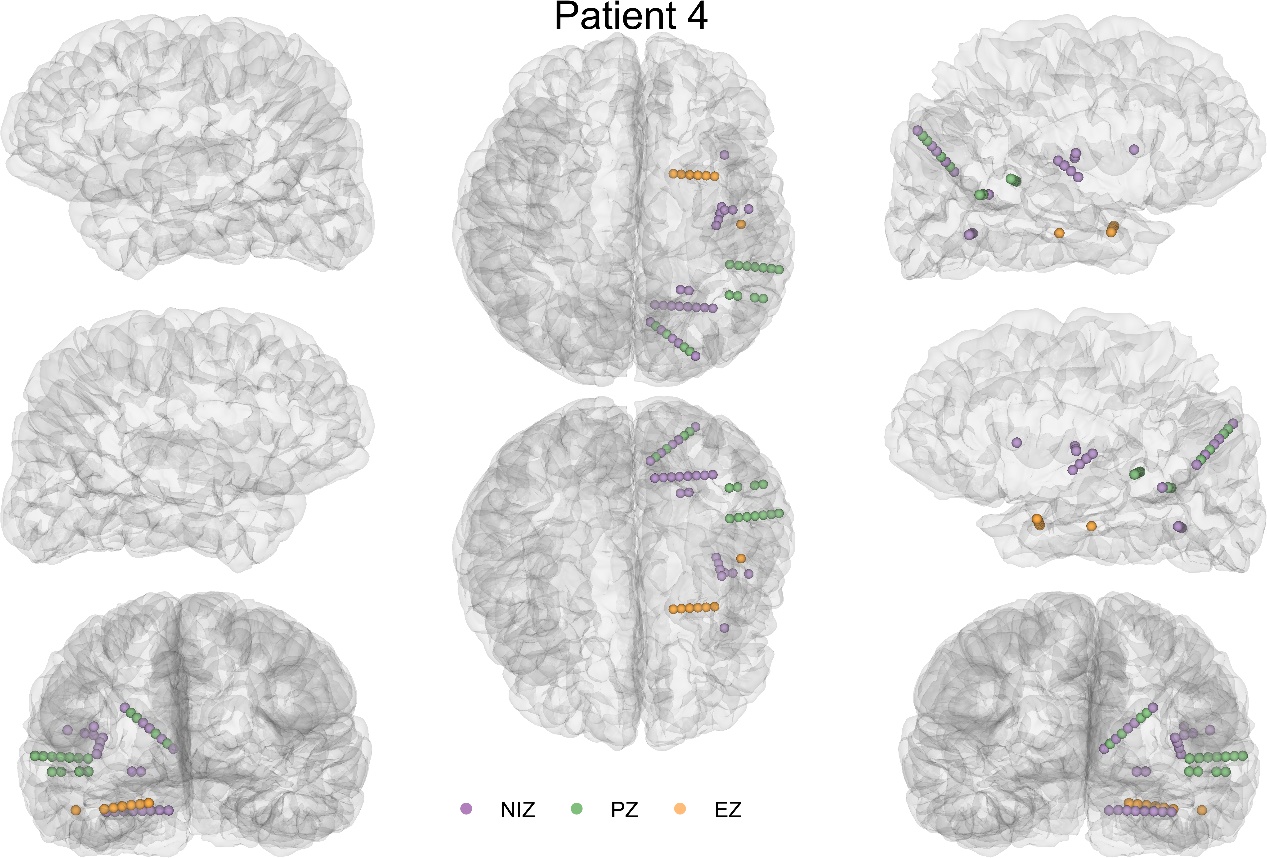


**Supplementary Figure 4.** **Electrode distributions of patient 4.**


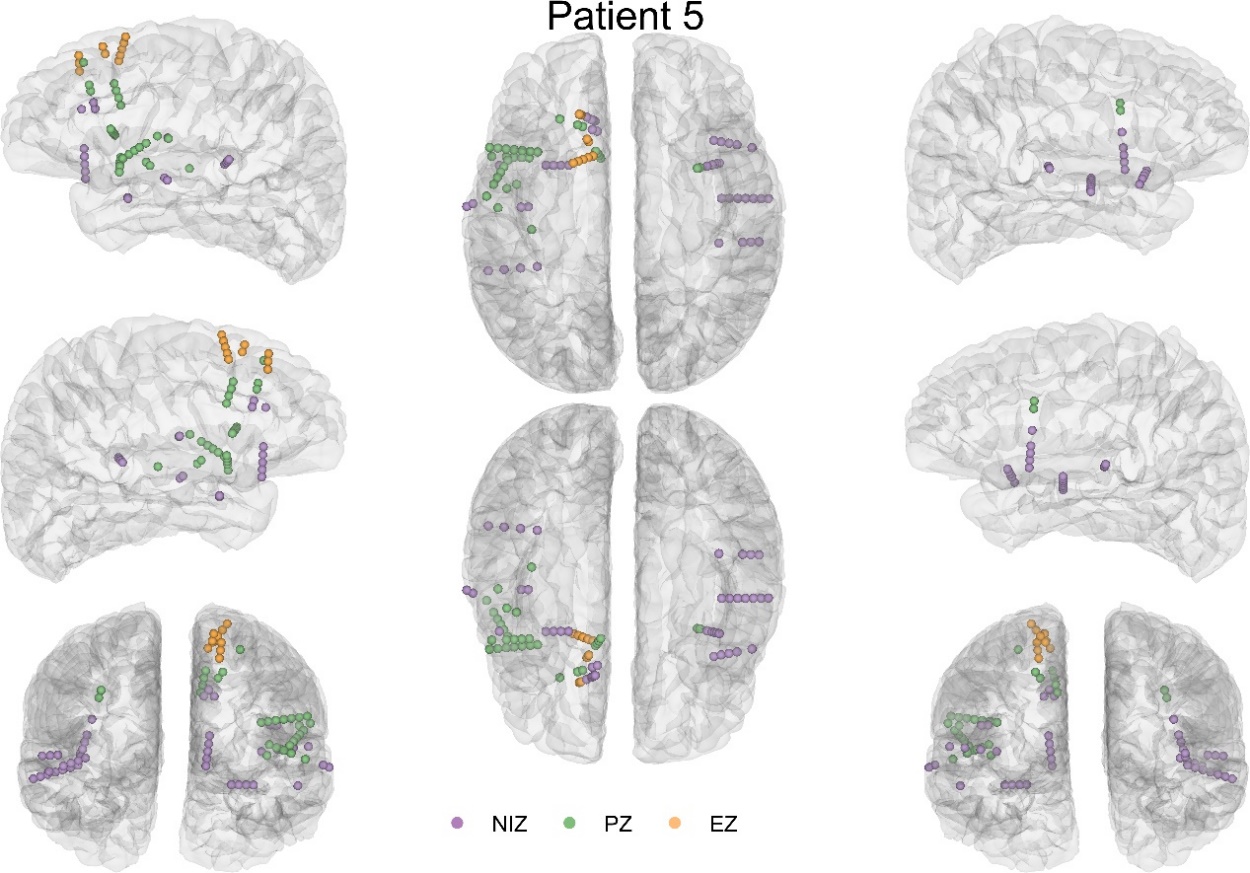


**Supplementary Figure 5.** **Electrode distributions of patient 5.**


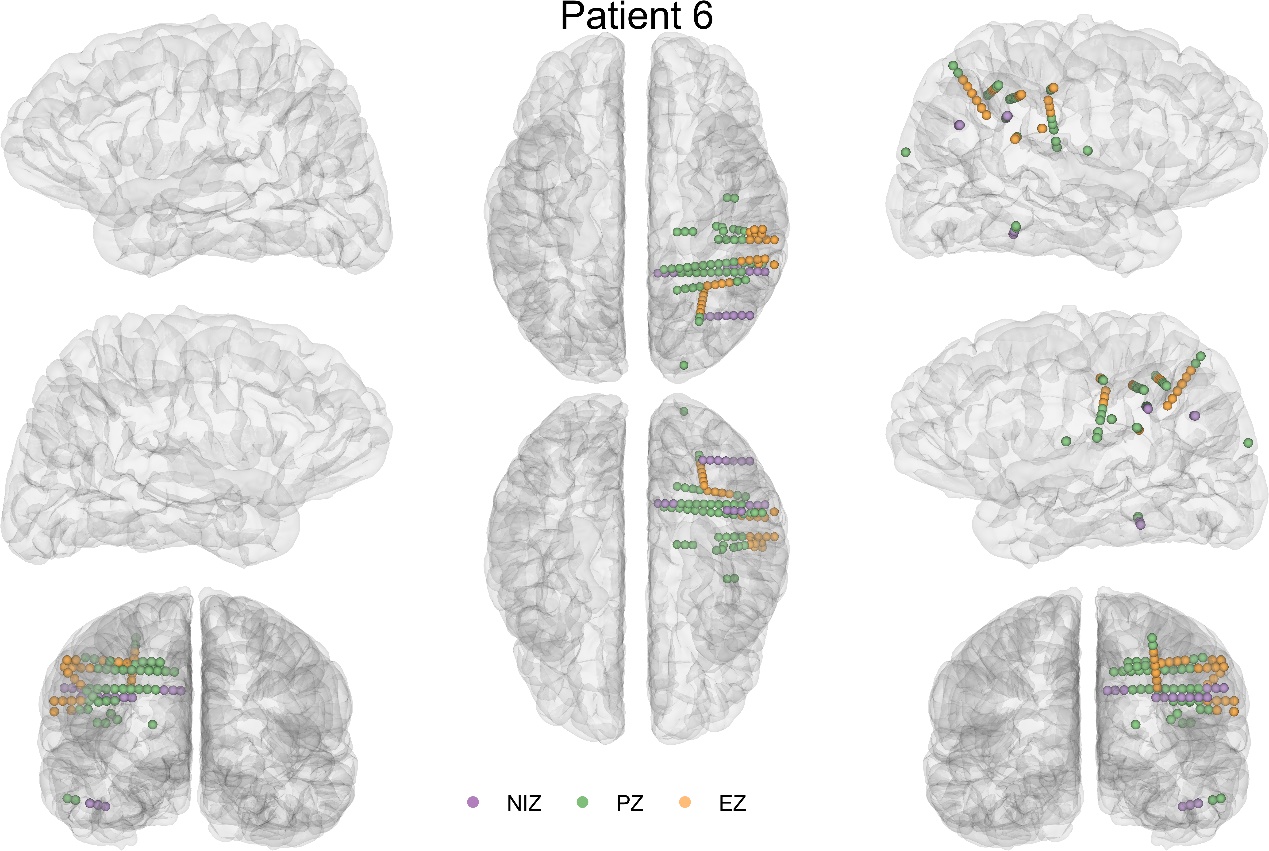


**Supplementary Figure 6.** **Electrode distributions of patient 6.**


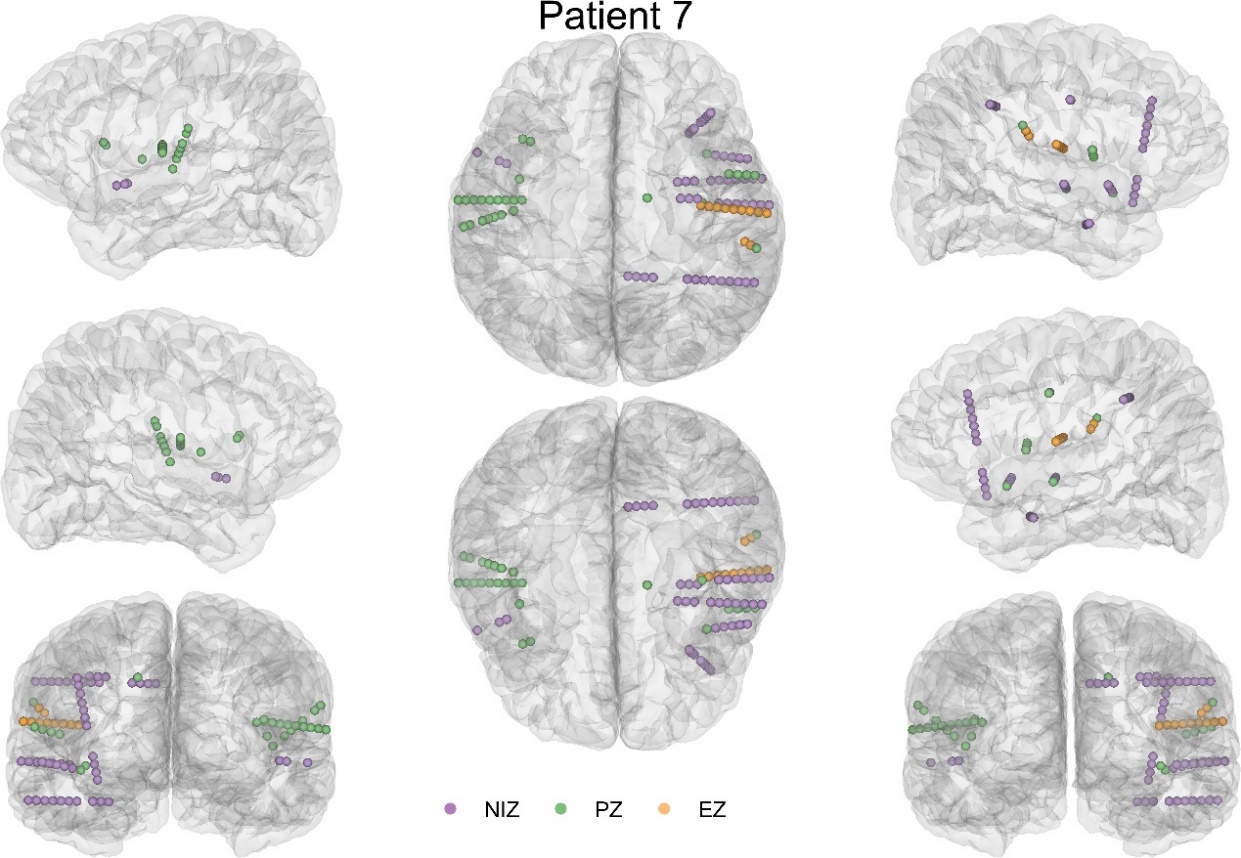


**Supplementary Figure 7.** **Electrode distributions of patient 7.**


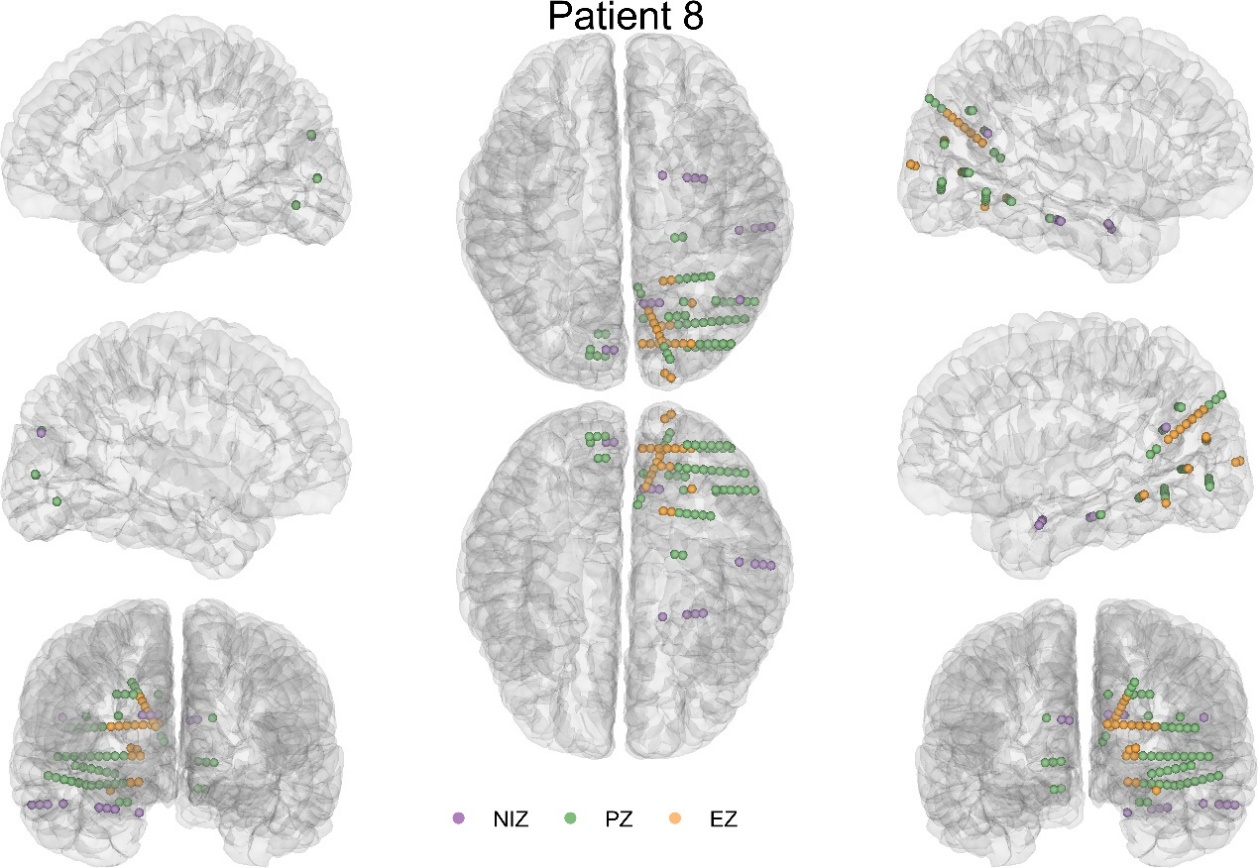


**Supplementary Figure 8.** **Electrode distributions of patient 8.**


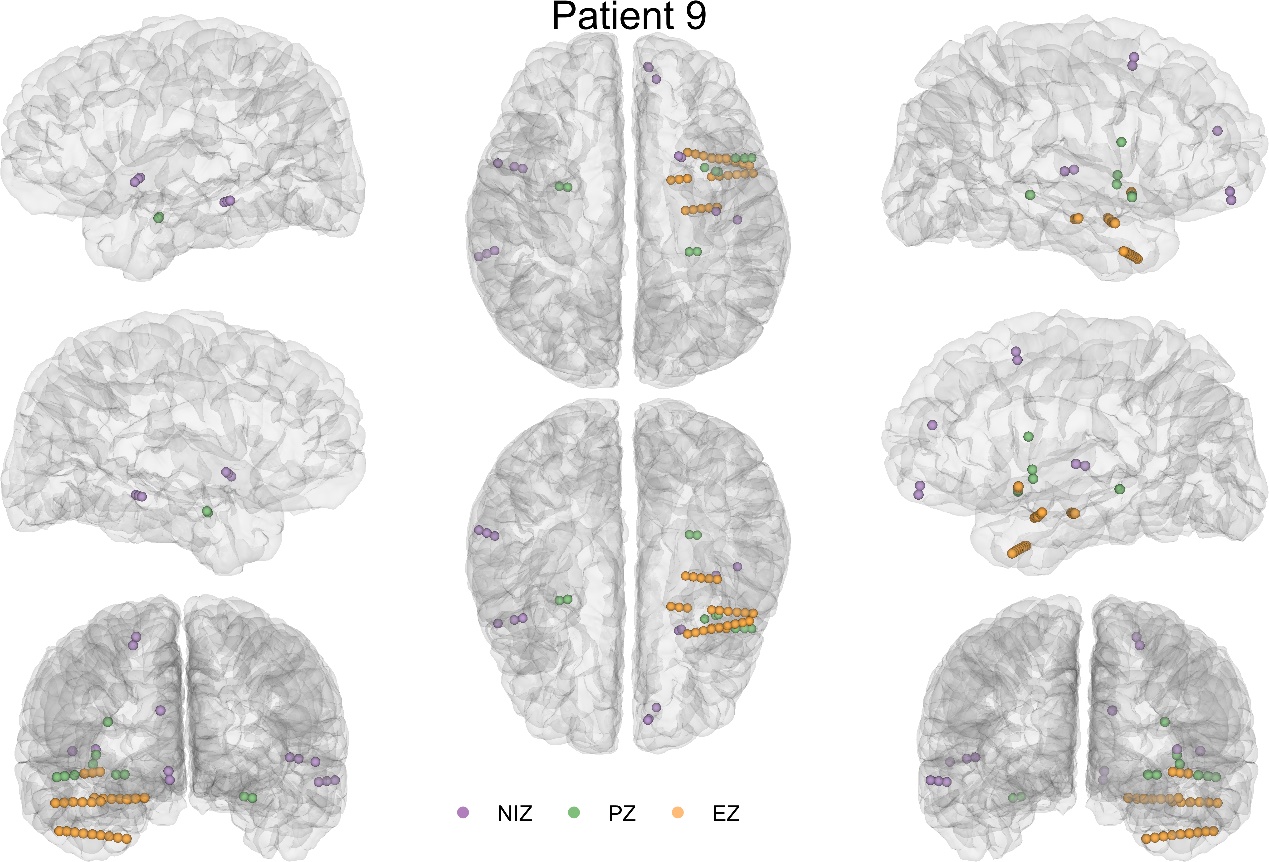


**Supplementary Figure 9.** **Electrode distributions of patient 9.**


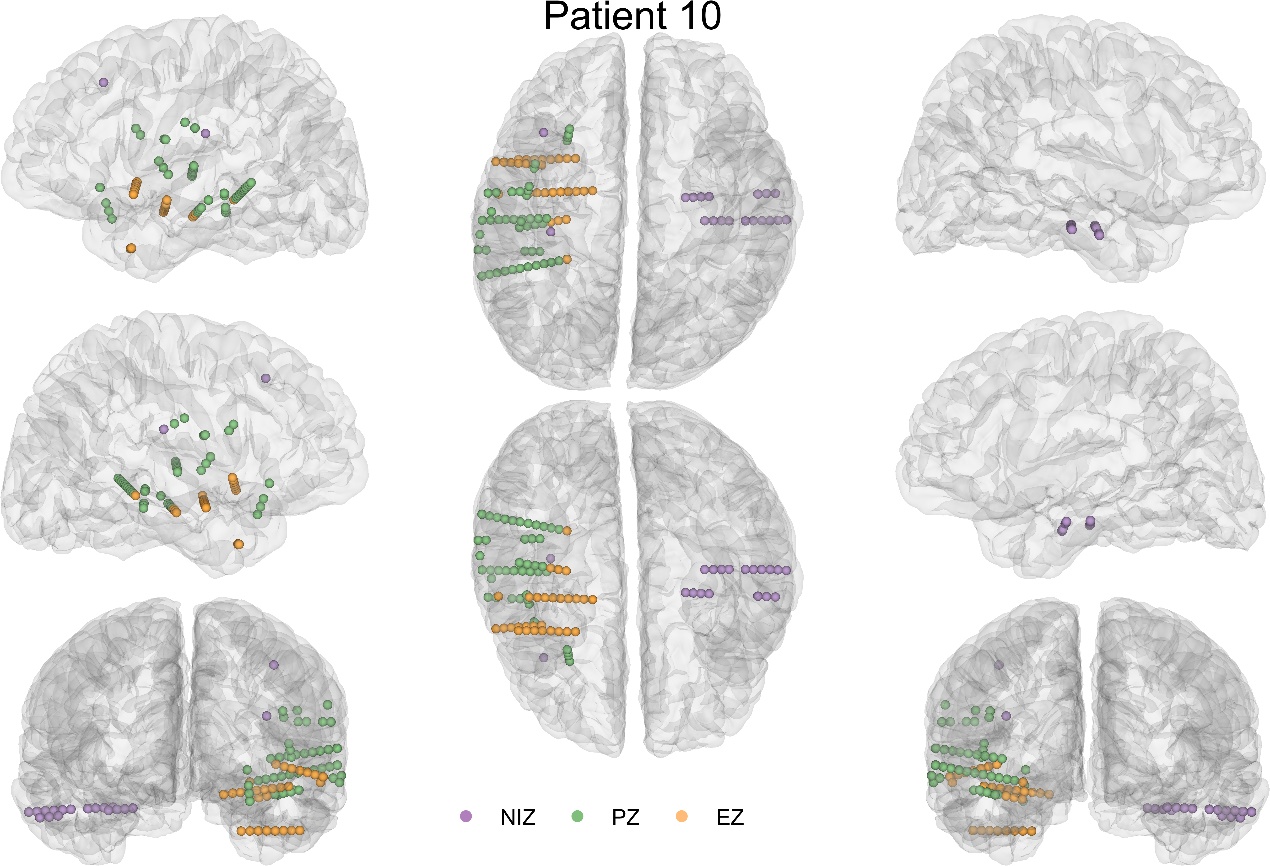


**Supplementary Figure 10.** **Electrode distributions of patient 10.**


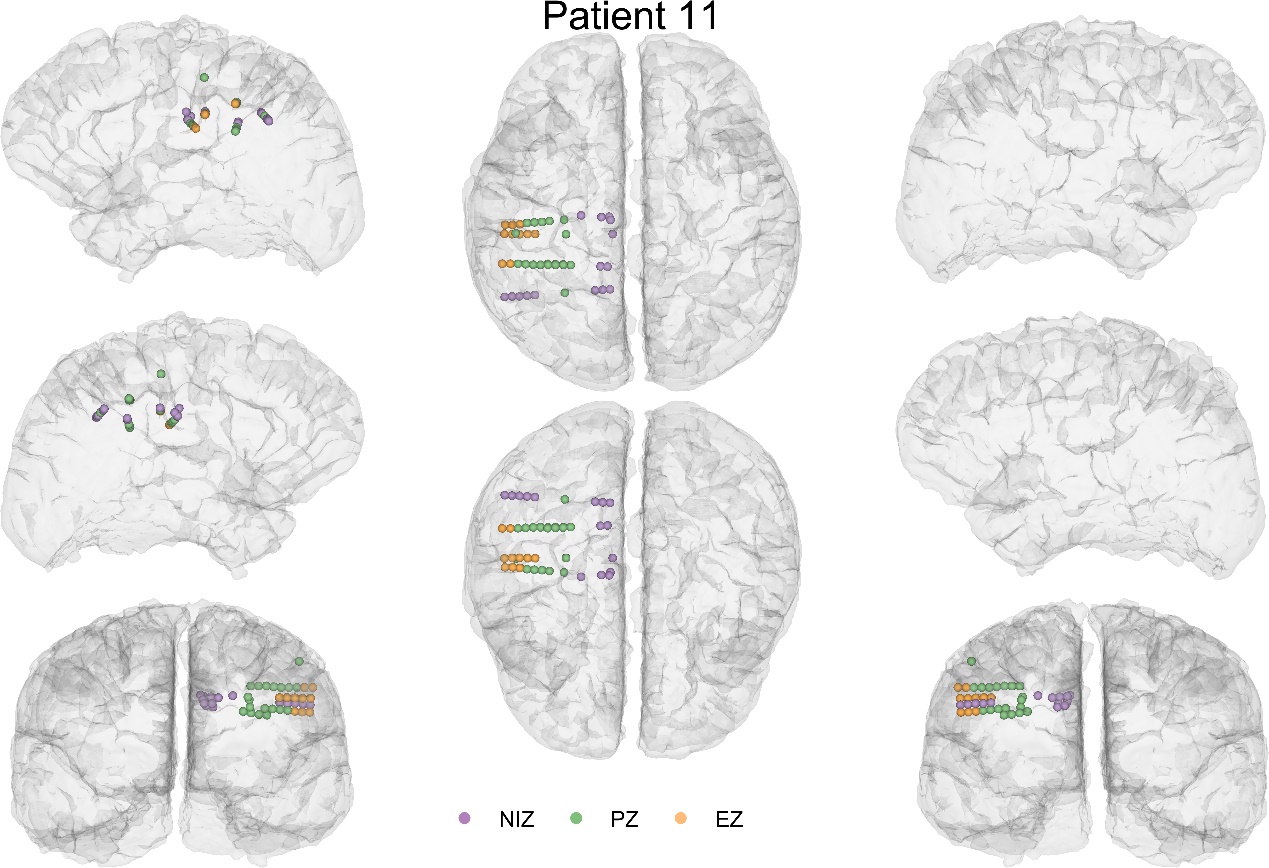


**Supplementary Figure 11.** **Electrode distributions of patient 11.**


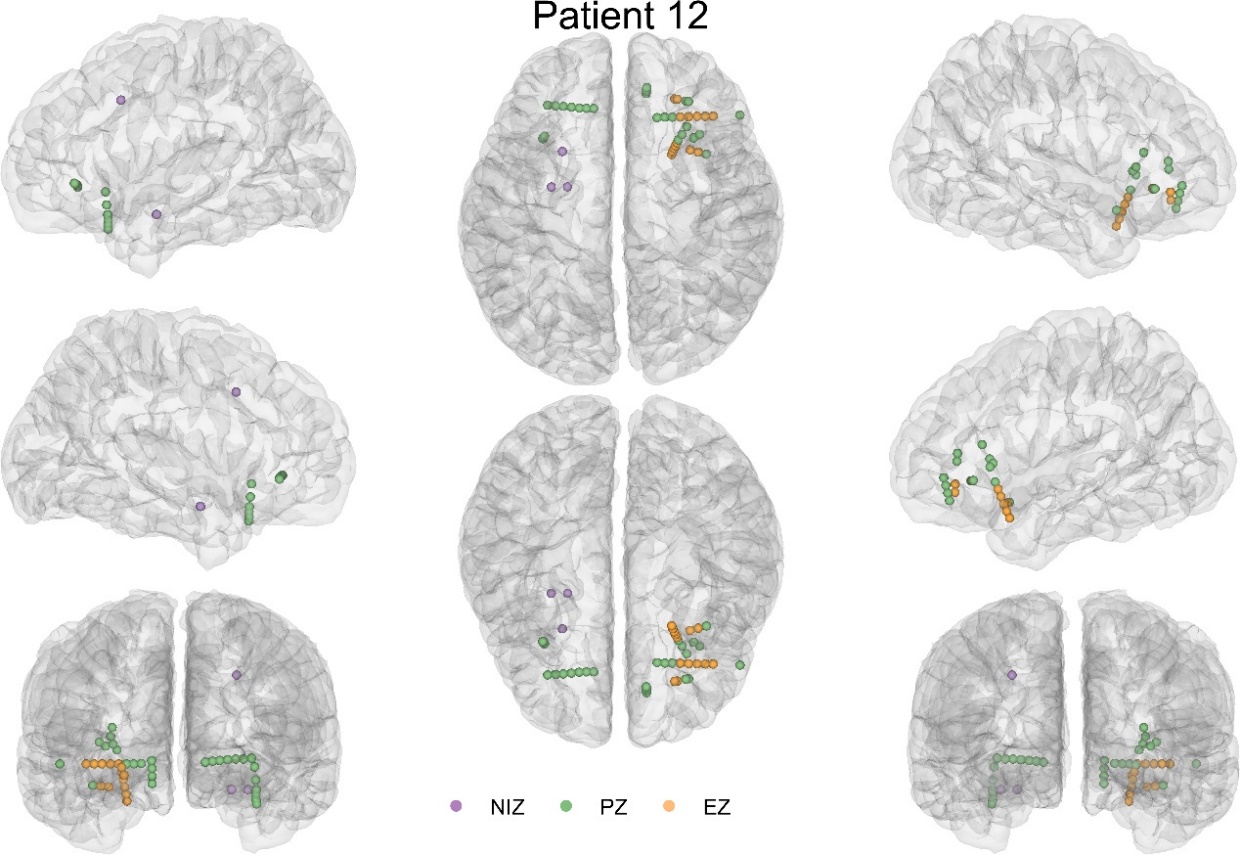


**Supplementary Figure 12.** **Electrode distributions of patient 12.**


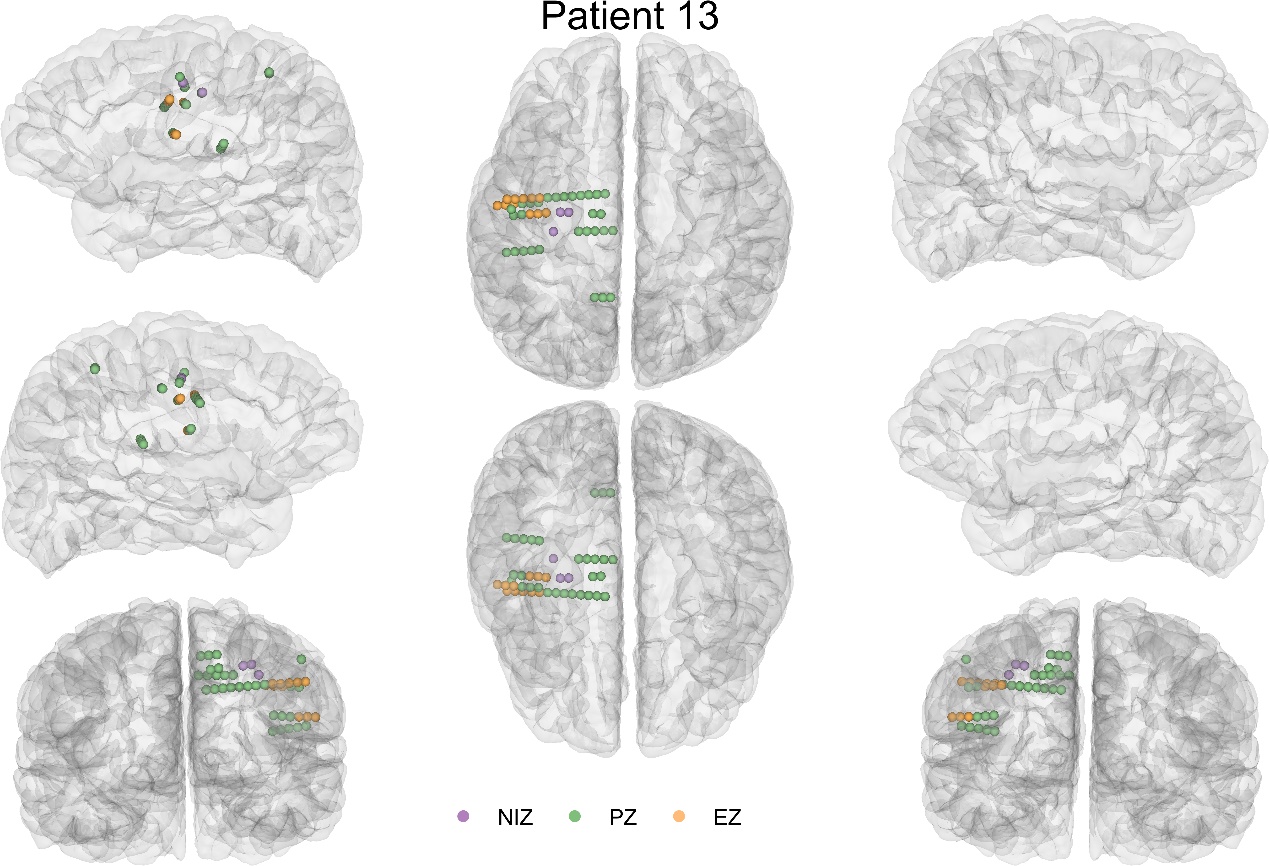


**Supplementary Figure 13.** **Electrode distributions of patient 13.**


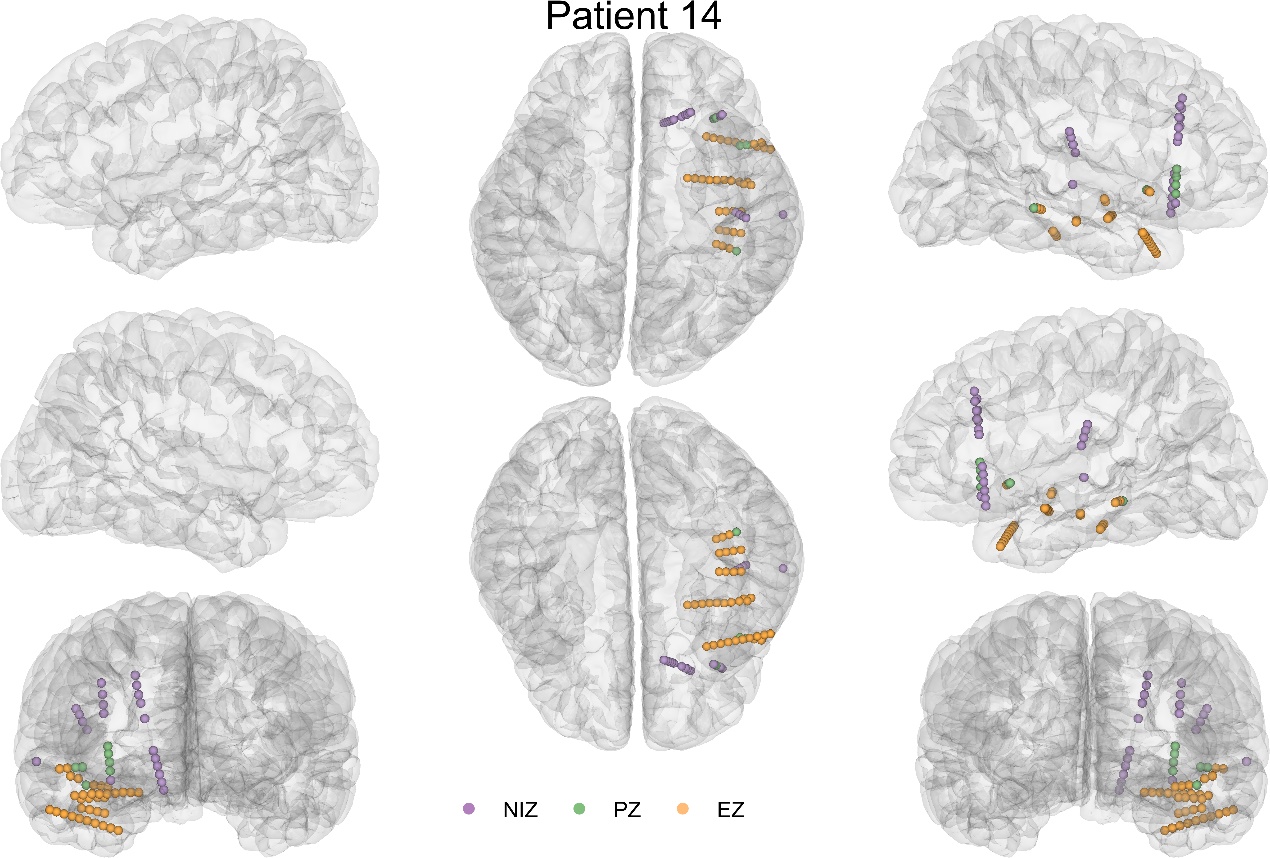


**Supplementary Figure 14.** **Electrode distributions of patient 14.**


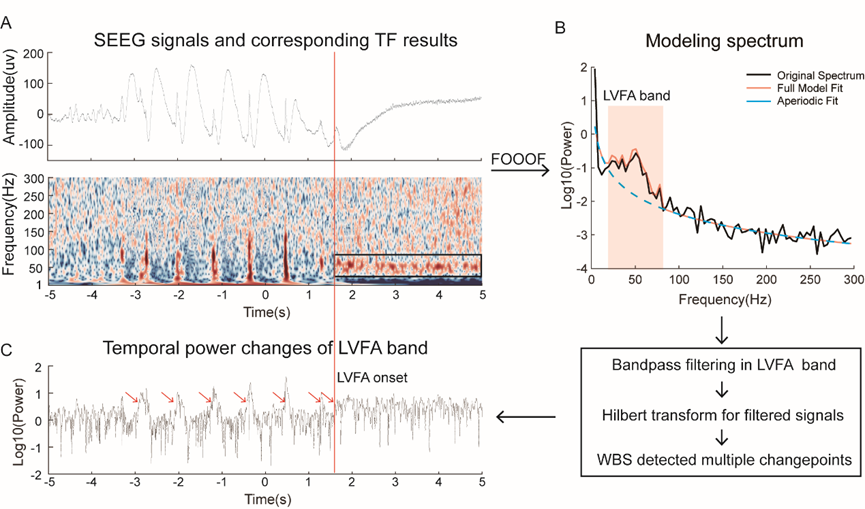


**Supplementary Figure 15.** **An example of the process for detecting the seizure onset for each electrode.** This example electrode is from one seizure in patient 10. (**A**) The top panel shows the SEEG signals from the pre-LVFA to ictal periods, with the zero-point representing the common seizure onset across all electrodes as marked by the physician. The time-frequency analysis results for the SEEG signals are displayed in the bottom panel, with the black box indicating the selected LVFA segment. (**B**) The selected LVFA segment underwent FOOOF fitting, resulting in the original spectrum (black line), the full modeled power spectrum (pink line) combining both periodic and non-periodic signals, and the aperiodic power spectrum (blue line). The LVFA frequency band range (pink shaded region) was determined by the parameters of the periodic spectrum. (**C**) The raw signal was then filtered with a bandpass FIR filter within this band, followed by a Hilbert transform to obtain temporal power changes. Multiple change points (red arrows) in power were detected using the WBS algorithm. The seizure onset (red lines) was determined by combining the occurrence of LVFA in the SEEG signals and the time-frequency analysis in panel A and the detected change points.

**
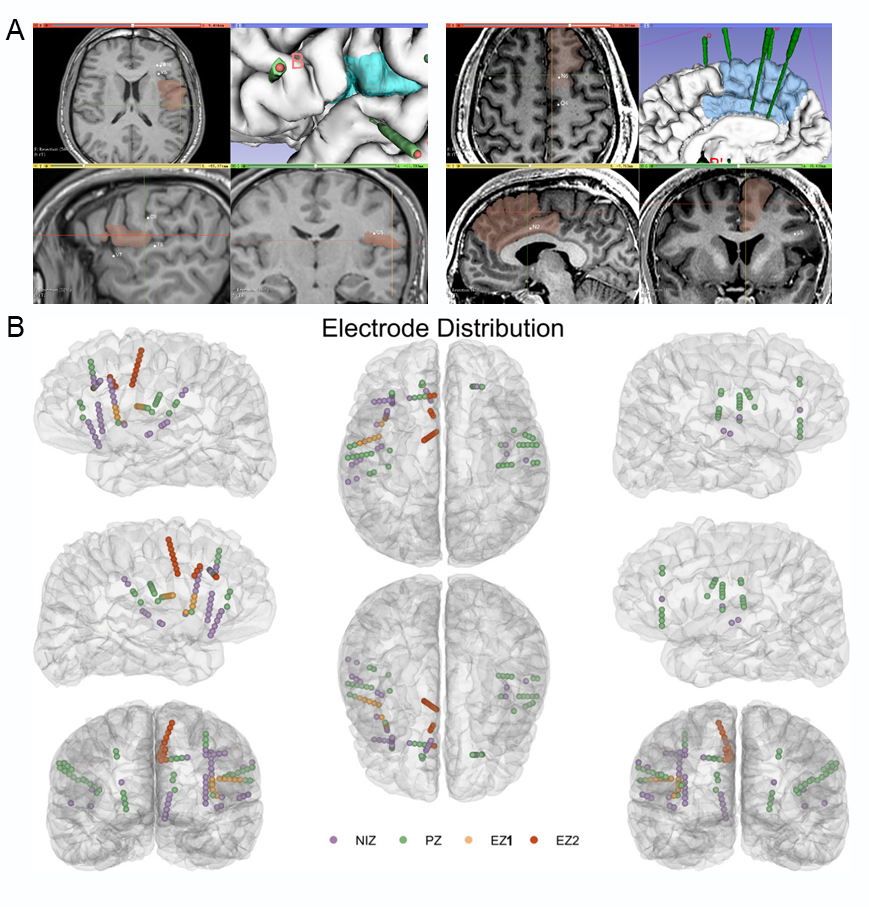
**

**Supplementary Figure 16.** **Surgical resection areas from the SEEG-guided surgery and electrode distributions.** (**A**) Left: Resection area based on the first SEEG evaluation in 2019 (marked in orange and blue), with a postoperative outcome of Engel class III. Right: Resection area based on the second SEEG evaluation in 2023 (marked in orange and blue), with a postoperative outcome of Engel class I. (**B**) Electrode distribution based on the first SEEG implantation.


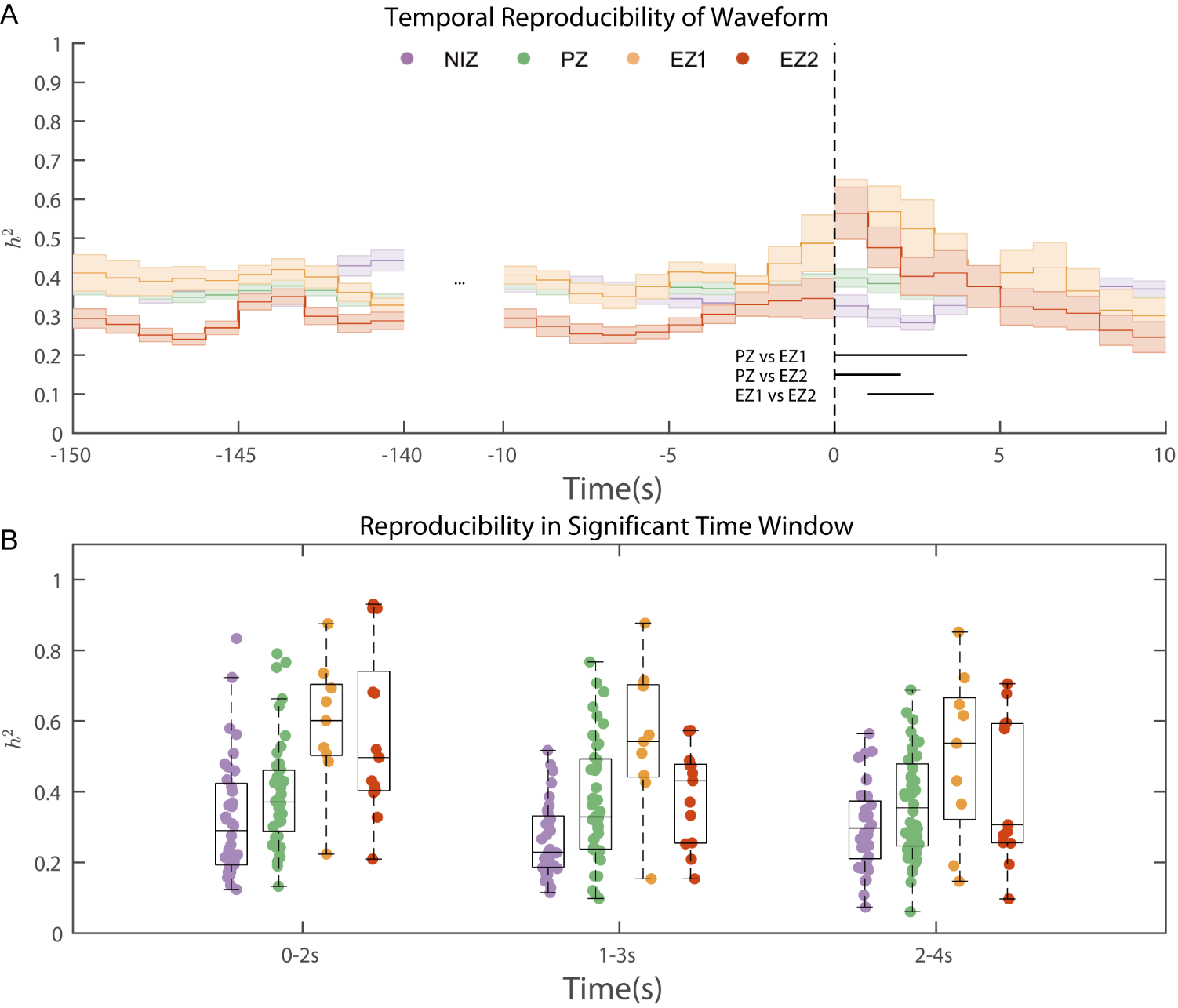


**Supplementary Figure 17.** **Between-seizure reproducibility of raw signals in NIZ, PZ, EZ1, and EZ2 during interictal, pre-LVFA, and ictal periods.** (**A**) Temporal evolution of raw SEEG signals reproducibility in each region, assessed using nonlinear correlation (*h²*) during the interictal, pre-LVFA, and ictal periods. The onset of LVFA is marked at time 0 (dotted vertical line). Horizontal black lines indicate the time windows with significant differences between regions (PZ vs EZ1, PZ vs EZ2, EZ1 vs EZ2) (*p < 0.05*). (**B**) Reproducibility during the significantly different time windows, showing box plots and scatter of electrode reproducibility in each region.


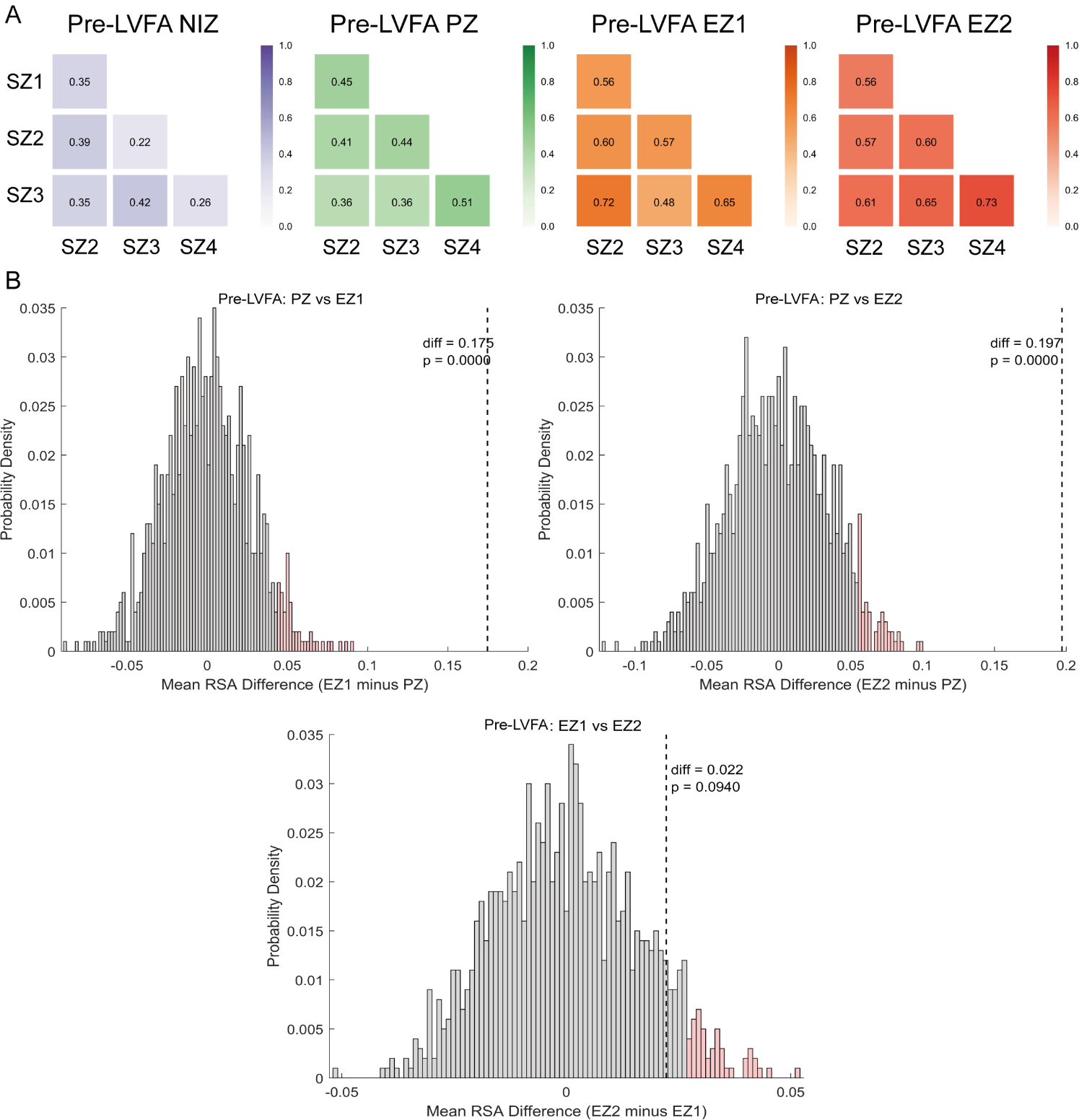


**Supplementary Figure 18.** **Between-seizure reproducibility of connectivity patterns within regions during pre-LVFA period.** (**A**) Representational similarity matrices (RSM) for within-region connectivity during the pre-LVFA period, showing the reproducibility of connectivity patterns across four seizures for six seizure pairs in the NIZ, PZ, EZ1, and EZ2 regions. Each matrix element represents the reproducibility between seizure pairs, with darker colors indicating higher reproducibility (numeric values annotated). (**B**) Statistical comparisons: Permutation distributions comparing the reproducibility of connectivity patterns between PZ vs EZ1, PZ vs EZ2, and EZ1 vs EZ2. The black dotted line indicates the observed value, with the corresponding mean difference and p-value shown. Pink areas highlight regions with p-values lower than 0.05.


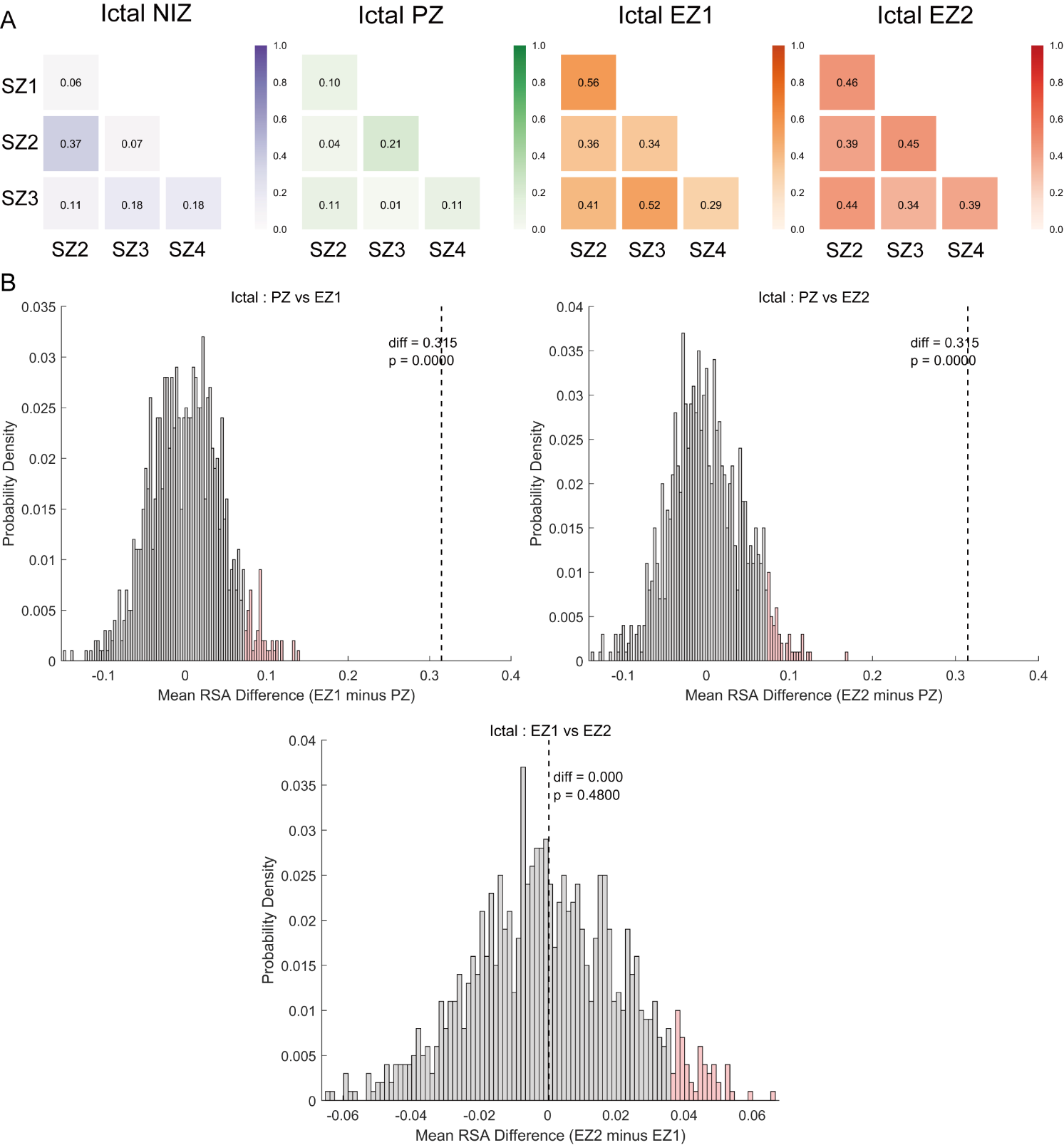


**Supplementary Figure 19.** **Between-seizure reproducibility of connectivity patterns within-regions during ictal period.** (**A**) Representational similarity matrices for within-region connectivity during the ictal period. (**B**) Statistical comparisons: Permutation distributions comparing the reproducibility of connectivity patterns between PZ vs EZ1, PZ vs EZ2, and EZ1 vs EZ2. The black dotted line indicates the observed value, with the corresponding mean difference and p-value shown. Pink areas highlight regions with p-values lower than 0.05.


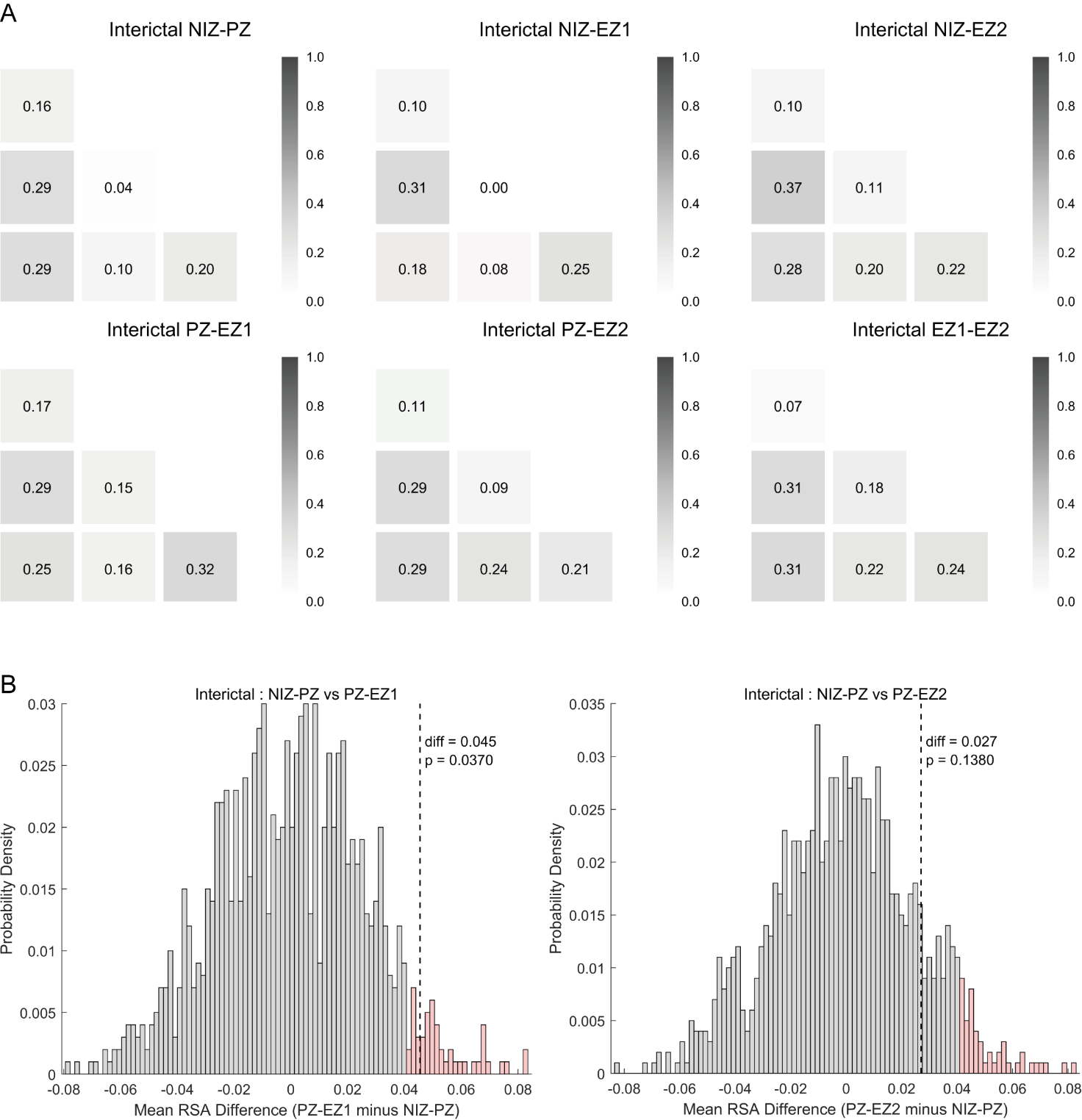


**Supplementary Figure 20. Between-seizure reproducibility of connectivity patterns between-regions during interictal period.** (**A**) Representational similarity matrices for between-region connectivity during the interictal period, showing the reproducibility of connectivity patterns across four seizures for six seizure pairs in the NIZ-PZ, NIZ-EZ1, NIZ-EZ2, PZ-EZ1, PZ-EZ2, and EZ1-EZ2. Each matrix element represents the reproducibility between seizure pairs, with darker colors indicating higher reproducibility (numeric values annotated). (**B**) Permutation distributions comparing the reproducibility of connectivity patterns between NIZ-PZ vs PZ-EZ1, NIZ-PZ vs PZ-EZ2. Electrode pairs from the two regions were randomly permuted (n = 1000), and the Kendall’ tau-a correlation coefficient was calculated for each permutation. The black dotted line indicates the observed value, with the corresponding mean difference and p-value shown. Pink areas highlight regions with p-values lower than 0.05.

**
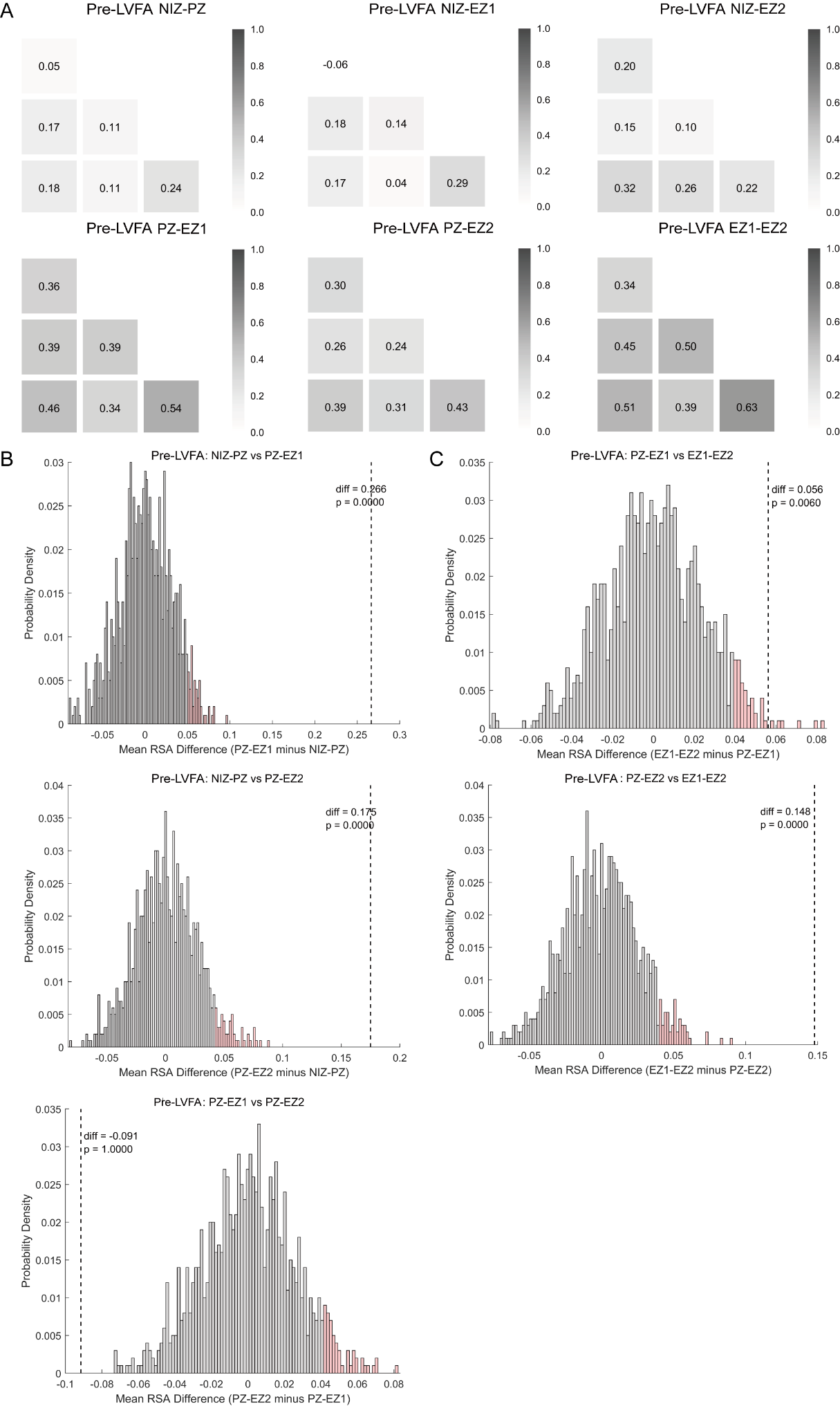
**

**Supplementary Figure 21 Between-seizure reproducibility of connectivity patterns between-regions during pre-LVFA period.** (**A**) Representational similarity matrices for between-region connectivity during the pre-LVFA period, with darker colors indicating higher reproducibility (numeric values annotated). (**B**) Permutation distributions comparing the reproducibility of connectivity patterns between NIZ-PZ vs PZ-EZ1, NIZ-PZ vs PZ-EZ2, and PZ-EZ1 vs PZ-EZ2. (C) Permutation distributions comparing the reproducibility of connectivity patterns between PZ-EZ1 vs EZ1-EZ2, and PZ-EZ2 vs EZ1-EZ2. The black dotted line indicates the observed value, with the corresponding mean difference and p-value shown. Pink areas highlight regions with p-values lower than 0.05.


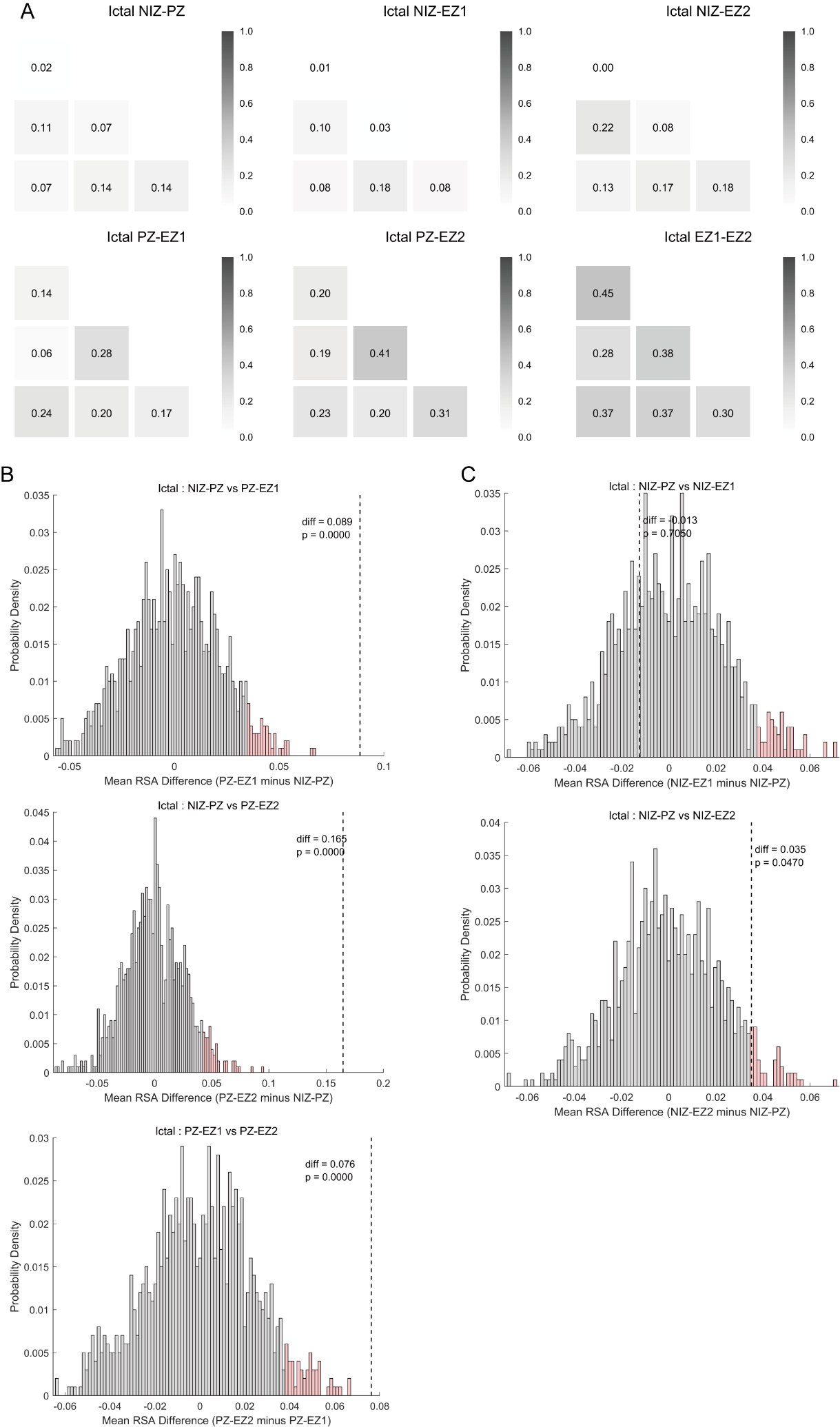


**Supplementary Figure 22. Between-seizure reproducibility of connectivity patterns between-regions during ictal period.** (**A**) Representational similarity matrices for between-region connectivity during the ictal period, with darker colors indicating higher reproducibility (numeric values annotated). (**B**) Permutation distributions comparing the reproducibility of connectivity patterns between NIZ-PZ vs PZ-EZ1, NIZ-PZ vs PZ-EZ2, PZ-EZ1 vs PZ-EZ2. (**C**) Permutation distributions comparing the reproducibility of connectivity patterns between NIZ-PZ vs NIZ-EZ1, and NIZ-PZ vs NIZ-EZ2. The black dotted line indicates the observed value, with the corresponding mean difference and p-value shown. Pink areas highlight regions with p-values lower than 0.05.


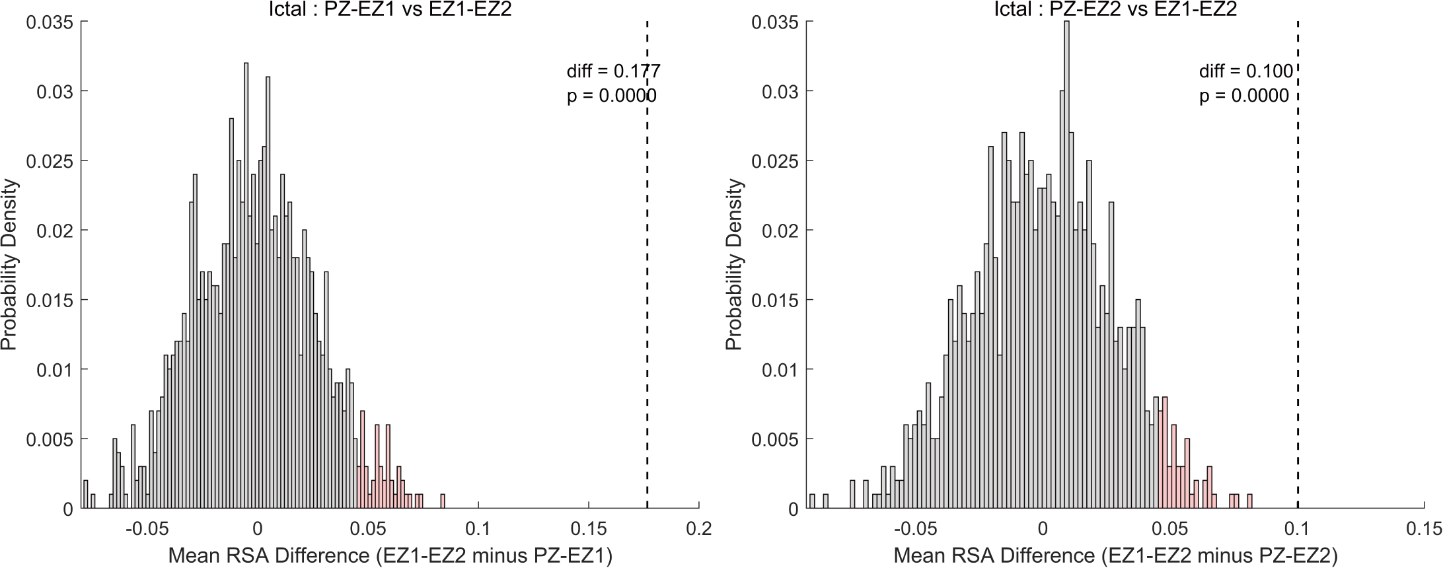


**Figure 23 Between-seizure reproducibility of connectivity patterns between-regions during ictal period.** Permutation distributions comparing the reproducibility of connectivity patterns between PZ-EZ1 vs EZ-EZ2, and PZ-EZ2 vs EZ1-EZ2. The black dotted line indicates the observed value, with the corresponding mean difference and p-value shown. Pink areas highlight regions with p-values lower than 0.05.
